# Characterising the shared genetic determinants of bipolar disorder, schizophrenia and risk-taking

**DOI:** 10.1038/s41398-021-01576-4

**Published:** 2021-09-08

**Authors:** Guy Hindley, Shahram Bahrami, Nils Eiel Steen, Kevin S. O’Connell, Oleksandr Frei, Alexey Shadrin, Francesco Bettella, Linn Rødevand, Chun C. Fan, Anders M. Dale, Srdjan Djurovic, Olav B. Smeland, Ole A. Andreassen

**Affiliations:** 1 NORMENT, Institute of Clinical Medicine, University of Oslo and Division of Mental Health and Addiction, Oslo University Hospital, 0407 Oslo, Norway; 2 Psychosis Studies, Institute of Psychiatry, Psychology and Neurosciences, King’s College London, London, UK; 3 Center for Bioinformatics, Department of Informatics, University of Oslo, Blindern, 0316 Oslo, Norway; 4 Department of Neurology, Division of Clinical Neuroscience, Oslo University Hospital, Oslo, Norway; 5 Multimodal Imaging Laboratory, University of California San Diego, La Jolla, CA 92093 USA; 6 Department of Radiology, University of California, San Diego, La Jolla, CA 92093 USA; 7 Department of Cognitive Science, University of California, San Diego, La Jolla, CA USA; 8 Department of Psychiatry, University of California, San Diego, La Jolla, CA USA; 9 Department of Medical Genetics, Oslo University Hospital, Oslo, Norway; 10 NORMENT, Department of Clinical Science, University of Bergen, Bergen, Norway

**Keywords:** Genetics, Comparative genomics

## Abstract

Increased risk-taking is a central component of bipolar disorder (BIP) and is implicated in schizophrenia (SCZ). Risky behaviours, including smoking and alcohol use, are overrepresented in both disorders and associated with poor health outcomes. Positive genetic correlations are reported but an improved understanding of the shared genetic architecture between risk phenotypes and psychiatric disorders may provide insights into underlying neurobiological mechanisms. We aimed to characterise the genetic overlap between risk phenotypes and SCZ, and BIP by estimating the total number of shared variants using the bivariate causal mixture model and identifying shared genomic loci using the conjunctional false discovery rate method. Summary statistics from genome wide association studies of SCZ, BIP, risk-taking and risky behaviours were acquired (*n* = 82,315–466,751). Genomic loci were functionally annotated using FUMA. Of 8.6–8.7 K variants predicted to influence BIP, 6.6 K and 7.4 K were predicted to influence risk-taking and risky behaviours, respectively. Similarly, of 10.2–10.3 K variants influencing SCZ, 9.6 and 8.8 K were predicted to influence risk-taking and risky behaviours, respectively. We identified 192 loci jointly associated with SCZ and risk phenotypes and 206 associated with BIP and risk phenotypes, of which 68 were common to both risk-taking and risky behaviours and 124 were novel to SCZ or BIP. Functional annotation implicated differential expression in multiple cortical and sub-cortical regions. In conclusion, we report extensive polygenic overlap between risk phenotypes and BIP and SCZ, identify specific loci contributing to this shared risk and highlight biologically plausible mechanisms that may underlie risk-taking in severe psychiatric disorders.

## Introduction

Schizophrenia (SCZ) and bipolar disorder (BIP) are severe mental disorders with overlapping clinical characteristics that are leading causes of morbidity and mortality worldwide [1]. With cardiovascular disease and suicide prominent in both [2–4], a better understanding of risk-taking behaviours such as smoking [5], substance use [6] and self-harm [7], could improve health outcomes [8]. Investigating genetic and neurobiological processes underlying the relationship between risk-taking, risky behaviours and SCZ and BIP may therefore offer novel opportunities for risk-stratification and intervention.

Risk-taking is defined as a willingness to engage in behaviours not only with potential reward but also potential harm [9]. Typified by certain risky behaviours such as overspending, it is a core feature of BIP and contributes to diagnostic criteria for the disorder [10, 11]. While a pronounced increase in risk-taking is associated with manic episodes [12], abnormalities in impulsivity, risk aversion and risk-seeking behaviour are also present as trait markers in euthymic people with BIP [13–15]. Additionally, mood stabilisers and anti-psychotics reduce impulsive and aggressive behaviour across a range of diagnoses, implying a shared neurobiological process to risk-taking beyond BIP [16, 17].

Unlike BIP, pronounced changes in risk-taking is not a core clinical feature of SCZ. However risky behaviours such as substance use, smoking and violence are more prevalent in individuals with SCZ than the general population [18–20]. Violence, in particular, is strongly associated with impulsivity, a neuropsychological domain closely related to risk-taking [20]. Findings from self-reports and neuropsychological measures are mixed. In one study, SCZ was not associated with self-reported risk perception [21] but Reddy et al. reported that individuals with SCZ were more risk averse than both individuals with BIP and healthy controls in a behavioural task [22]. Moreover, impulsivity has been shown to be both increased and decreased in subjects with SCZ, likely dependent on the subtype of SCZ and the presence or absence of psychosis [22, 23].

Various experimental studies have attempted to delineate the neurobiology underlying risk-taking, BIP and SCZ. Impulsivity and dysfunctional reward processing in BIP and SCZ have been associated with deficits in the prefrontal cortex on both functional and structural measures [23–26], loss of grey matter in the anterior cingulate cortex (ACC) [27, 28] and reduced white matter integrity in the cingulum and frontal lobes [29, 30]. Moreover, dopaminergic neurotransmission in the mesolimbic reward system has been strongly implicated in risk-taking and risky behaviours [31], demonstrated by the association between impulse control disorders and dopamine agonists [32].

A recent genome-wide association study (GWAS) of risk-taking and risky behaviours in over 1 million participants has provided new insights into their genetic architecture [33]. Using questionnaire measures for the propensity to take risks and the first principal component of four risky behaviours, 99 risk-taking loci were identified, 46 of which were shared with risky behaviours, implicating glutamatergic and GABAergic neurotransmission [33]. Significant positive genetic correlations were reported between risk-taking and BIP (*r*_g_ = 0.21) and SCZ (*r*_g_ = 0.17), suggesting a shared genetic basis [33, 34]. However, little is known about the individual genetic loci driving these findings. Moreover, genetic correlation does not provide a complete representation of the shared genetic architecture between two phenotypes [35]. This is demonstrated by recent evidence of overlap between similar complex polygenic phenotypes with multiple shared loci, but a mixture of concordant and opposite effects leading to minimal genetic correlation [36, 37]. Additionally, both BIP and SCZ are highly heritable, with SNP-based heritability estimates ranging from 20 to 40% [38–46]. An improved understanding of this genetic component will provide insights into their aetiology, and identify novel targets for prevention and treatment [47].

We therefore employed the bivariate causal mixture model (MiXeR) [43] and the conjunctional false discovery rate method (conjFDR) [36, 48] to large-scale GWASs for BIP [38] and SCZ [40] together with risk-taking and risky behaviours [33] in order to a) quantify the total number of shared variants regardless of effect direction, b) identify individual loci driving the phenotypic overlap between risk, SCZ and BIP and c) leverage polygenic overlap to boost statistical power to identify novel loci associated with SCZ and BIP.

## Methods

### Samples

We acquired GWAS summary statistics from recent publications. The SCZ sample comprised 35,476 cases with SCZ and 46,839 controls [40]. The BIP sample comprised 41,917 cases and 371,549 controls [49]. The risk-taking cohort comprised 466,751 individuals [33]. All samples were of European descent. Four lakhs thirty-one thousand one hundred and twenty-six of the risk-taking sample were derived from the UK Biobank (UKB), and were assessed by a single yes/no item asking, “would you describe yourself as someone who takes risks?” The remaining 35,445 comprised ten individual cohorts. Risky behaviours were measured by calculating the first principal component of four risky behaviours in UKB (automobile speeding propensity, alcoholic drinks per week, number of sexual partners and ever smoker”) (*n* = 315,894). These items were chosen because they have been shown to correlate with self-reported risk-taking in independent samples [50–54], represent distinct domains of risk-taking (namely driving, alcohol drinking, smoking and sexual behaviours), and they were available in the entire sample [33]. For further details see supplementary methods and the original publications [33, 38, 40]. The Regional Committee for Medical Research Ethics—Southeast Norway has evaluated the current protocol, and found that no additional institutional review board approval was necessary as no individual data were used. Relevant ethics committees approved all primary GWASs, and all participants provided informed consent [33, 39, 49].

### Data analysis

We employed MiXeR to quantify polygenic overlap between each psychiatric disorder and risk phenotype [43]. A bivariate Gaussian mixture model using GWAS summary statistics was constructed to estimate the total number of shared and phenotype-specific variants that explains 90% of SNP heritability in each phenotype. Model fit is based on likelihood maximisation of signed test statistics (GWAS *z*-scores) evaluated by the Akaike Information Criterion (AIC), and demonstrated with predicted versus observed conditional quantile–quantile (Q–Q) plots. See supplementary methods and supplementary Fig. 1 for further information. We also calculated LD-score regression genetic correlation [42].

Conditional Q–Q plots were constructed to visualise cross-trait enrichment between each pair of phenotypes. Conditional Q–Q plots compare the association between individual SNPs and a primary phenotype (e.g., SCZ) as a function of their association with a secondary phenotype (e.g., risk-taking). Cross-trait enrichment is present if there are successive leftward deflections from the expected Q–Q plot under the null hypothesis (i.e., that there are no SNPs associated with the primary phenotype), signifying a higher proportion of SNPs associated with the primary phenotype as the strength of association with the secondary phenotype increases [48].

To identify individual SNPs jointly associated with both phenotypes, we employed conjFDR analysis using a threshold of conjFDR <0.05 [48]. Further details of the conjFDR analysis can be found in supplementary methods and prior publications [36, 48, 55, 56]. ConjFDR is also able to identify novel associations with each phenotype beyond genome-wide significance due to the boost in power from the cross-trait analysis.

### Genomic loci definition

Independent significant SNPs, lead SNPs, candidate SNPs, and genomic loci margins were defined using the FUMA protocol (http://fuma.ctglab.nl/). See supplementary methods for further information. Novel loci were determined by cross-referencing identified loci with previous GWASs and other relevant studies [33, 37–40, 48, 55, 57–66].

### Functional annotation

Putative causal genes were mapped to lead SNPs using three gene-mapping methodologies: 1) positional mapping which matches SNPs to their nearest genes, 2) expression quantitative trait loci (eQTL) mapping that identifies genes whose expression is associated with the SNPs’ allelic variation, 3) chromatin interaction mapping that matches SNPs to genes with which they are predicted to interact based on chromatin structure [67]. We conducted differential gene expression analyses using GTEx eQTL data, Gene Ontology gene-set analyses using FUMA [68, 69], pathway analyses using Consensus PathDB [70] and spatiotemporal gene expression analysis of mapped genes using BrainSpan RNA sequencing data [71–73]. All analyses were corrected for multiple comparisons using Bonferroni correction. Further details are provided in Supplementary methods.

All code is publicly available at https://github.com/precimed.

## Results

### Estimating total genetic overlap

**Fig. 1:**
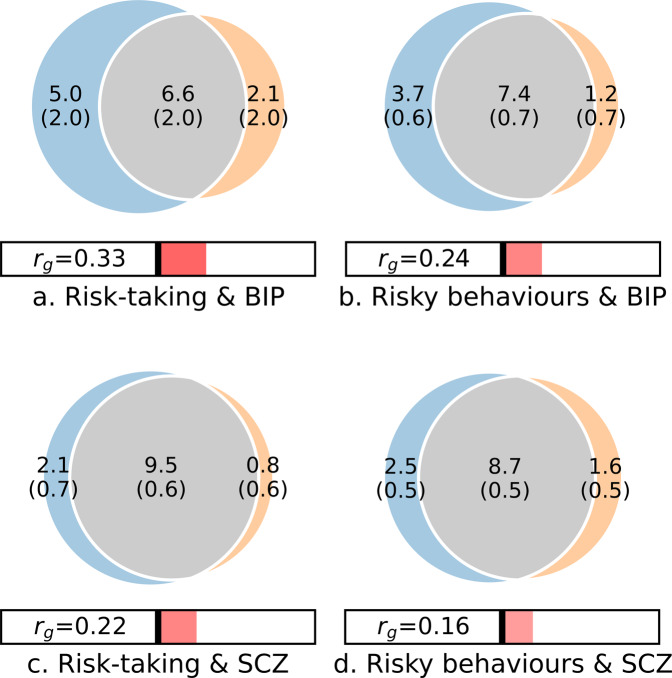
Genome-wide genetic overlap between risk phenotypes, bipolar disorder and schizophrenia. MiXeR analysis for **a** bipolar disorder (BIP) and risk-taking, **b** BIP and risky behaviours, **c** schizophrenia (SCZ) and risk-taking, and **d** SCZ and risky behaviours. Venn diagrams representing the unique and shared variants associated with risk-taking and risky behaviours and each of SCZ and BIP. Polygenic overlap is represented in grey, Risk/risky behaviours in blue and BIP/SCZ in orange. The numbers indicate the estimated quantity of variants in thousands per component that explains 90% of SNP heritability for each phenotype (standard error in parentheses). The size of the circle reflects the extent of polygenicity for each trait.

MiXeR demonstrated substantial polygenic overlap between BIP, SCZ, and each of risk-taking and risky behaviours, beyond that captured by genetic correlation (Fig. 1). Of a total of 8.6–7 K variants estimated to influence BIP, 6.6 K (SD = 2.0 K, 77%) and 7.4 K (SD = 0.7, 85%) were also estimated to influence risk-taking and risky behaviours, respectively. Similarly, of a total of 10.2–10.3 K variants predicted to influence SCZ (N.B. estimates of polygenicity for individual traits can differ between analyses due to the random-pruning process), 9.6 K (SD = 0.5 K, 94%) and 8.8 K (SD = 0.7, 85%) were predicted to influence risk-taking and risky behaviours, respectively. We also found highly significant positive genetic correlations between all phenotypic pairings (risk-taking/BIP: *r*_g_ = 0.33, *p* = 2.35e^−31^; risky behaviours/BIP: *r*_g_ = 0.24, *p* = 7.50^−16^; risk-taking/SCZ: *r*_g_ = 0.22, *p* = 7.41e^−16^; risky behaviours/SCZ: *r*_g_ = 0.16, *p* = 2.08e^−10^), replicating previous findings [8, 33]. Despite this, the extent of the overlap in relation to the size of the genetic correlations indicated a mixture of shared variants with concordant and discordant effects on each pair of phenotypes. Accordingly, MiXeR estimated that 57–72% (SD 0.5–12%) of shared SNPs had concordant effects (Supplementary Table 1). MiXeR also illustrated all four phenotypes’ extensive polygenicity. Risk-taking and risky behaviours were estimated to be particularly polygenic (11.5 K and 11.1 K variants respectively), helping to explain why smaller proportions of risk-taking and risky behaviour-associated variants were predicted to influence mental disorders (57–83%). Model fit was adequate (further details in the Supplementary results).

### Visualising cross-trait enrichment

**Fig. 2:**
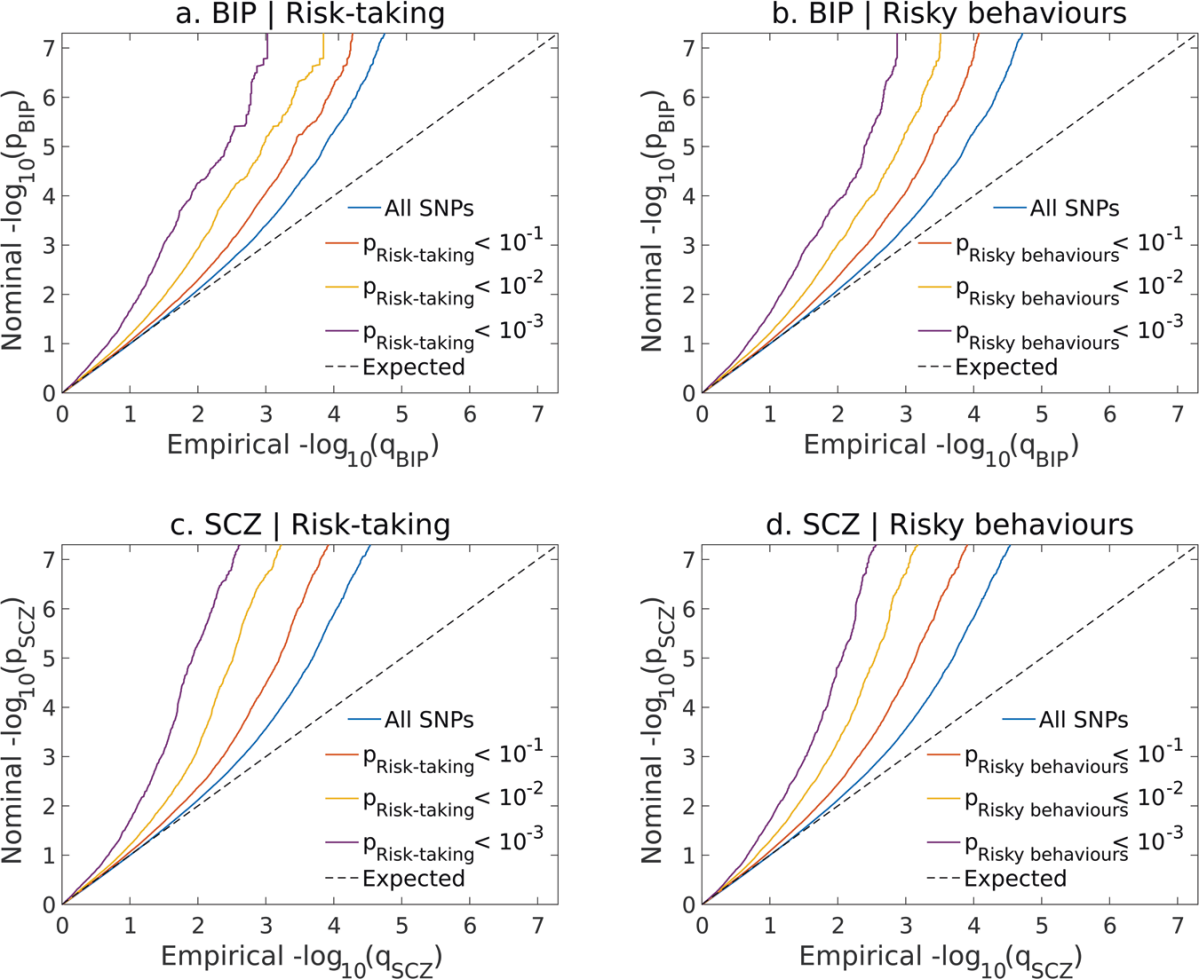
Polygenic enrichment of SNPs associated with bipolar disorder (BIP) and schizophrenia (SCZ) dependent on their association with risk taking and risky behaviours. Visualising cross-trait enrichment between risk phenotypes, bipolar disorder and schizophrenia. Conditional Q–Q plots of nominal versus empirical −log10 *p* values in BIP (**a**, **b**) and SCZ (**c**, **d**) above the threshold of *p* < 5 × 10^−8^ as a function of the significance of their association with risk-taking (a./c.) and risky behaviours (RiskyBehav) (b./d.) at the level of *p* < 0.1, *p* < 0.01 and *p* < 0.001, respectively.

Conditional Q–Q plots demonstrated step-wise increments in SNP enrichment for SCZ and BIP as a function of the strength of their association with risk-taking and risky behaviours (Fig. 2), and for risk-taking and risky behaviours as a function of their association with SCZ and BIP (Supplementary Fig. 2). This further demonstrated cross-trait enrichment between phenotypes.

### Shared loci between SCZ, BIP and risk-taking

**Fig. 3:**
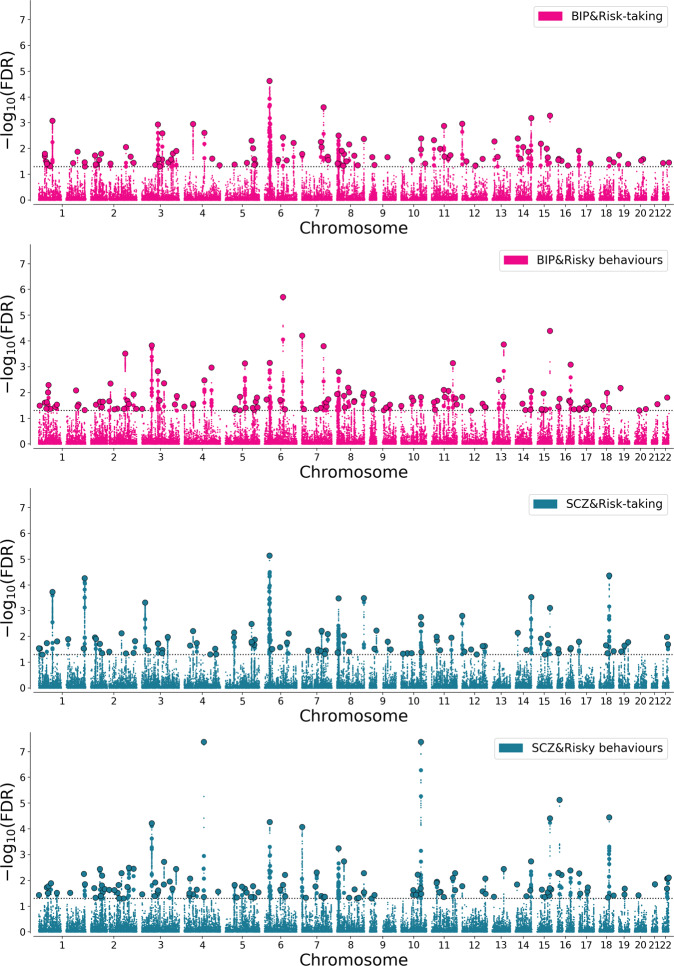
Discovery of genetic loci jointly associated with risk phenotypes, bipolar disorder and schizophrenia. Manhattan plots showing the −log10 transformed conjFDR values for **A** bipolar disorder (BIP) and risk-taking, **B** BIP and risky behaviours, **C** schizophrenia (SCZ) and risk-taking and **D** SCZ and risky behaviours risk for each SNP (y-axis) against chromosomal position (x-axis). The dotted line represents the threshold for significant association (conjFDR  <  0.05). Black circles represent lead SNPs.

At a threshold of conjFDR<0.05, we identified 106 and 131 loci jointly associated with BIP and each of risk-taking and risky behaviours respectively, 98 of which were novel in BIP and 31 were overlapping across both risk phenotypes (Fig. 3, Table 1, and Supplementary Table 2). 88% (93/106) and 85% (111/131) shared the same direction of effect on BIP and risk-taking and risky behaviours, respectively, in line with the positive genetic and MiXeR predictions. With regards SCZ, there were 100 and 129 loci jointly associated with SCZ and each of risk-taking and risky behaviours, respectively, of which 38 were novel in SCZ (Fig. 3 and Supplementary Table 3). Furthermore, 37 loci were overlapping across both risk-taking and risky behaviours analyses (Table 2), and 18 were also overlapping with loci associated with BIP and both risk phenotypes (Table 1 and Supplementary Tables 2–3). 76% (76/100) and 74% (96/129) of all lead SNPs had the same direction of effect on SCZ and each of risk-taking and risky behaviours, respectively.

**Table 1:** Ten genomic loci jointly associated with bipolar disorder (BIP) and risk-taking which physically overlapped with loci jointly associated with BIP and risky behaviours at conjunctional FDR (conjFDR) < 0.05.

Chr	Min–max BP	Lead SNPs	ConjFDR	Direction of effect	Novel in BIP	Novel in risk-taking	Mapped genes
1	33,761,014–33,878,554	rs12138864^a^	0.019	+	×	×	*PHC2*^b^
1	44,029,353–44,196,945	rs2367724	0.039	+	×	Yes	*MED8*^b^*, KDM4A*^b^*, ARTN*^b^
1	61,671,468–61,743,723	rs182823	0.048	+/−	×	Yes	*NFIA*^b^
1	205,646,278–205,799,987	rs823130	0.013	+	×	×	*NUCKS*^b^*, RAB7L1*^b^*, SLC41A1*^b^*, PM20D1*^b^
1	243,503,764–243,579,112	rs4146671	0.034	+	Yes	×	*SDCCAG8*^b^
2	104,056,454–104,380,545	rs1433309	0.038	+	×	Yes	–
2	225,334,070–225,467,840	rs2047134	0.036	+	Yes	×	*CUL3*^b^
3	84,841,679–85,789,644	rs9831123^a^	0.001	+	×	×	*CADM2*^b^
3	94,027,330–94,229,614	rs4857445	0.050	+	×	Yes	–
4	46,191,375–46,361,545	rs535066	0.001	+	Yes	×	*GABRA2*^b^
4	106,061,534–106,417,856	rs2647256^a^	0.002	+	×	×	*TET2*^b^
5	49,442,082–49,775,649	rs20158778^a^	0.042	+	Yes	Yes	–
5	152,181,095–152,329,944	rs10053762	0.026	+	×	Yes	–
6	25,182,925–31,870,326^c^	rs7746199	0.000	+	×	×	*BTN3A2, BTN3A3, PRSS16*^b^*, POM121L2, ZNF184, ZSCAN23*
6	98,310,291–98,792,109	rs1487445	0.004	+	×	×	–
7	1,860,733–2,110,850	rs11768212	0.016	+	×	Yes	*MAD1L1*^b^
7	114,940,147–115,117,353	rs10251192	0.000	+	Yes	×	–
7	140,116,033–140,182,514	rs80274100	0.028	+	×	Yes	*RAB19*^b^*, MKRN1*^b^
8	9,306,087–10,006,664	rs73207111	0.003	+	×	×	–
8	38,014,429–38,310,910	rs11777067	0.016	+	x	Yes	*LSM1*^b^
8	65,437,506–65,499,486	rs7813444^a^	0.007	+	Yes	×	*DDHD2*^b^*, PPAPDC1B*^b^*, WHSC1L1*^b^*, FGFR1*^b^
8	92,976,563–93,180,965	rs28716374	0.019	+	×	Yes	–
8	143,363,229–143,404,118	rs34853464	0.004	+	×	Yes	*RUNX1T1*^b^
10	56,629,277–56,706,723	rs9888039	0.028	+	Yes	Yes	*TSNARE1*^b^
10	104,571,436–104,962,011	rs7085104^a^	0.011	+/−	×	Yes	*PCDH15*^*2*^
11	13,230,633–13,350,131	rs10082688	0.005	+	×	×	*ARL3*^b^*, WBP1L*^b^*, AS3MT*^b^*, CNNM2*^b^*, NT5C2*^b^
12	2,285,731–2,420,526	rs61909095	0.001	+	×	×	*CACNA1C*^b^
12	108,609,634–108,629,780	rs3764002	0.025	+	Yes	×	*WSCD2*^b^
14	103,849,715–104,528,302	rs12892189	0.001	+	×	×	*KLC1, PPP1R13B*^b^*, TDRD9*
15	33,761,014–33,878,554	rs2071382	0.001	+	×	Yes	*FURIN*^b^*, FES*^b^
16	44,029,353–44,196,945	rs6500948	0.028	+	Yes	Yes	*RBFOX1*^b^

*Chr* chromosome, *min–max BP* minimum–maximum base pair position, *ConjFDR* conjFDR value of lead SNP, Direction of effect -“+” concordant effect directions of lead SNPs, “+/−” discordant effect direction of lead SNPs on BIP and risk-taking, Novel BIP/risk-taking: “Yes” indicates novel loci, Mapped genes genes mapped to lead SNPs for BIP and risk-taking.

^a^Lead SNPs that were also lead SNPs in the risky behaviour loci. For corresponding BIP and risky behaviour loci please refer to Supplementary Table 3.

^b^Genes that were also mapped to lead SNPs for BIP and risky behaviours.

^c^This locus is within the major histocompatibility complex. Given the complex LD in this region, gene mapping strategies are not reliable. Mapped genes are provided but should be interpreted with caution.

**Table 2:** Thirty-seven genomic loci jointly associated with schizophrenia (SCZ) and risk-taking which physical overlapped with loci jointly associated with SCZ and risky behaviours at conjunctional FDR (conjFDR) < 0.05.

Chr	Min-max BP	Lead SNPs	ConjFDR	Direction of effect	Novel in SCZ	Novel in risk-taking	Mapped genes
1	3,121,877–3,147,290	rs7518852^a^	0.028	+	×	Yes	*PRDM16*^b^
1	43,982,527–44,145,130	rs673253	0.018	+	×	Yes	*TMEM125, TIE1, MED8*^b^*, PTPRF*^b^
1	243,281,617–243,645,203	rs10803138	<0.001	+	×	×	*SDCCAG8*
2	48,176,656–48,707,841	rs138659652	0.019	+	Yes	Yes	*MSH6, FBXO11*
2	97,593,614–98,332,858	rs115507803	0.038	+	×	Yes	*FAHD2B*
2	162,798,581–162,891,848	rs6707646	0.007	+/−	×	Yes	*SLC4A10*
2	225,334,070–225,467,840	rs2047134	0.043	+	×	×	*CUL3*^b^
3	84,841,679–84,961,810	rs1598080	0.018	+	×	×	–
3	86,011,109–86,192,846	rs58783194^a^	0.019	+/−	×	Yes	–
4	31,162,143–31,201,229	rs7669969^a^	0.022	+	Yes	×	–
4	46,191,375–46,361,545	rs10805144	0.006	+	Yes	×	*GABRA2*^b^
4	66,258,524–66,534,629	rs13124331^a^	0.018	+	×	×	*EPHA5*^b^
5	45,411,676–50,161,698	rs13168483	0.007	+	×	Yes	*EMB*^b^
6^c^	25,684,606–29,283,672	rs6923811	<0.001	+	×	×	*BTN3A2, POM121L2*
7	114,940,147–115,113,279	rs4275159^a^	0.039	+	Yes	×	–
7	121,952,981–122,008,804	rs988720^a^	0.048	+	×	Yes	*FEZF1*^b^*, CADPS2*^b^
8^c^	8,088,230–12,203,305	rs9329221	<0.001	+	×	×	*MSRA*^b^*, FAM167A*
8	38,014,429–38,310,910	rs7845911	0.009	+	×	Yes	*LSM1*^b^*, BAG4*^b^*, DDHD2*^b^*, PPAPDC1B*^b^
8	65,437,964–65,498,165	rs6996198^a^	0.039	+	×	×	–
8	143,276,606–143,404,118	rs13281016	<0.001	+	×	Yes	*TSNARE1*^b^
9	26,447,292–27,111,268	rs56409537	0.030	+	×	Yes	–
10	104,546,183–105,165,256	rs12416687	0.002	+/−	×	Yes	*ARL3*^b^*, WBP1L*^b^*, BORCS7*^b^*, AS3MT*^b^
10	106,417,957–106,560,225	rs12761679	0.003	+	×	Yes	*SORCS3*^b^
11	27,581,265–27,742,447	rs6265	0.010	+	×	Yes	*BDNF*
11	28,375,949–28,580,338	rs12419325	0.014	+	×	Yes	*METTL15*
12	2,285,731–2,420,526	rs61909095	0.002	+	×	×	*CACNA1C*^b^
12	108,609,634–108,629,780	rs3764002	0.024	+/−	×	×	*WSCD2*^b^
14	78,625,653–78,659,721	rs7153461^a^	0.033	+/−	Yes	Yes	–
14	103,849,715–104,537,680	rs6576006	<0.001	+	×	×	*KLC1*^b^*, PPP1R13B*^b^*, TDRD9*
15	63,460,009–63,547,859	rs6494397	0.050	+/−	Yes	Yes	*RPS27L*
15	78,714,561–78,926,726	rs12442456	0.009	+/−	×	Yes	*IREB2, ADAMTS7*^b^
15	91,403,674–91,443,059	rs2071382	0.001	+	×	Yes	*FES*^b^*, FURIN*^b^
16	7,324,960–7,417,203	rs8046401	0.031	+	×	Yes	*RBFOX1*^b^
16	71,355,142–71,396,661	rs3826248	0.031	+	×	×	*CMTR2, DHODH, DHX38*^b^
18	53,183,396–53,477,999	rs1382119	<0.001	+	×	×	–
19	30,982,165–31,052,954	rs10421376	0.022	+/−	×	Yes	*ZNF536*^b^
22	41,485,593–42,396,890	rs75843224	0.010	+/−	×	Yes	*EP300*

*Chr* chromosome, *min–max BP* minimum–maximum base pair position, *ConjFDR* conjFDR value of lead SNP, Direction of effect “+” concordant effect directions of lead SNPs, “+/−” discordant effect direction of lead SNPs on SCZ and risk-taking, Novel SCZ/risk-taking: “Yes” indicates novel loci; Mapped genes—genes mapped to lead SNPs for SCZ and risk-taking.

^a^Lead SNPs that were also lead SNPs in the risky behaviour loci. For corresponding SCZ and risky behaviour loci please refer to Supplementary Table 2.

^b^Genes that were also mapped to lead SNPs for SCZ and risky behaviours.

^c^These loci reside within regions possessing complex LD structure and so gene mapping strategies are not reliable. Mapped genes are provided but should be interpreted with caution.

### Functional annotation

We mapped 142 and 177 protein-coding genes to lead SNPs for BIP and each of risk-taking and risky behaviours respectively (Supplementary Tables 4 and 5). Thirty-nine genes were mapped to both sets of lead SNPs, including the calcium channel *CACNA1C* and the synaptic cell adhesion molecule *CADM2* [74]. Expression of mapped genes was significantly enriched in 30 and 25 tissues for risk-taking and risky behaviours, respectively (Supplementary Figs. 3 and 4). The amygdala, hippocampus, anterior cingulate and multiple basal ganglia structures were among the top ten tissues for both analyses. Gene-set analysis identified 50 gene-sets significantly enriched with mapped genes for risk-taking and BIP, 14 of which were specific to neuronal structure (Supplementary Table 6) and pathway analysis identified 19 overrepresented pathways (Supplementary Table 7). Regarding risky behaviours and bipolar, 36 gene-sets were enriched, 14 of which were also enriched in risk-taking and bipolar, including seven of the neuronal structure gene-sets. Thirty-seven pathways were over-represented with mapped genes for risky behaviours and BIP, five of which were associated with GABA-ergic neurotransmission. Further, “twelve loci associated with ADHD”, “protein–protein interactions at synapse” and “nicotine addiction” were also present in BIP and risk-taking (Supplementary Tables 6 and 7).

With regards SCZ, we mapped a total of 131 and 181 genes to lead SNPs associated with SCZ and each of risk-taking and risky behaviours, respectively (supplementary Tables 8 and 9). Twenty-nine were mapped to lead SNPs from both analyses, including the GABA receptor subunit *GABRA2* [75] and *EPHA5*, a tyrosine kinase implicated in neurodevelopment (Table 2) [76]. When testing differential tissue expression of mapped genes, three structures in the basal ganglia (caudate, putamen and nucleus accumbens) were the most significantly enriched tissues for both analyses (Supplementary Figs. 5 and 6). Gene-set analysis identified 34 gene-sets enriched with mapped genes for risk-taking and SCZ, and 54 for risky behaviours and SCZ (Supplementary Table 10). Among these, 19 were common to both risk phenotypes, 14 of which were related to neuron development, structure or function. Pathway analysis of the same sample of genes identified 18 and 30 pathways significantly overrepresented with mapped genes for SCZ and each of risk-taking and risky behaviours, respectively. A single pathway, “Twelve loci atssociated with ADHD”, was common to all four analyses (Supplementary Table 11). Further functional annotations and spatiotemporal gene expression analyses are presented in Supplementary results, Supplementary Tables 12–15, and Supplementary Figs. 7 and 8.

## Discussion

In this analysis of GWAS summary statistics, we reveal extensive polygenic overlap between mental disorders and risk phenotypes beyond genetic correlation and identify and characterise independent genomic loci underlying this overlap. Using MiXeR, we first estimated that 77–94% of all BIP or SCZ influencing variants also influence risk-taking and risky behaviours, despite moderate positive genetic correlations. This has implications for how the genetic risk for mental-health-related traits is conceptualised, suggesting most variants influence multiple traits with few phenotype-specific variants. We next identified 206 genomic loci jointly associated with BIP and risk phenotypes and 192 associated with SCZ and risk phenotypes using conjFDR. Of these, 98 were novel in BIP and 38 were novel in SCZ, contributing to ongoing efforts to reveal the missing heritability of SCZ and BIP. Furthermore, 74–88% of lead SNPs had concordant effects on mental disorders and risk phenotypes, in line with positive genetic correlations. Finally, we highlight the role of multiple cortical and sub-cortical brain structures and neuronal development, structure, and function in risk phenotypes and both disorders. These findings may lead to the new mechanistic hypotheses, the identification of novel treatment targets and enable risk stratification of risk-taking and risky behaviours in severe psychiatric disorders.

Using MiXeR, we demonstrated that most variants associated with BIP and SCZ also influence risk-taking and risky behaviours, despite genetic correlations between 0.16–0.30 [43]. While this may be surprising, genetic correlation provides a summary measure between −1 and 1 of the correlation of effect sizes. This means that mixtures of variants with concordant and opposite effects “cancel each other out”, resulting in a genetic correlation of 0. The extensive overlap is therefore compatible with these modest genetic correlation estimates since MiXeR predicted 57–72% of shared variants had concordant effect sizes. While genetic correlation is useful to understand how the overall genetic risk for one phenotype covaries with the genetic risk for another, uncovering the fraction of overlapping and unique variants provides another dimension to the characterisation of shared genetic architecture. Indeed, these findings are consistent with a growing body of evidence suggesting that, despite differing genetic correlations, there is widespread polygenic overlap of a similar extent across a diverse range of mental-health-related phenotypes, including almost total overlap between SCZ and educational attainment [35, 77]. Taken together, these findings have implications for how the genetic risk of complex polygenic traits, like BIP and SCZ, is conceptualised. If polygenic overlap is the norm, then each risk variant is likely to be highly non-specific and influence multiple diverse traits. This would imply that it is, in fact, the specific distribution of effect sizes and effect directions, along with the interaction between different risk variants, that differentiates risk for a specific phenotype rather than a specific set of variants [35].

Nonetheless, the higher proportion of concordant lead SNPs and moderate positive genetic correlations indicate a genetic basis to the increased risk-taking and risky behaviours observed in BIP, suggesting that risk-taking may represent a genetically influenced endophenotype for BIP [15]. In contrast, risk-taking has been reported to be both increased and decreased in SCZ [22], while risky behaviours, such as smoking and violence, are increased. Our findings therefore suggest that there is a similar genetic tendency for risk-taking and risky behaviours in SCZ, as with BIP. This indicates that conflicting findings in SCZ are likely to be influenced by methodological and environmental factors, such as the use of neuropsychiatric measures that correlate poorly with self-report measures [78], antipsychotic medication use [21] and cognitive symptoms [79] rather than differences in genetic influences. This also suggests that differences in risk-taking and risk-behaviour may be a trait-marker in SCZ as in BIP, although this requires further investigation [13–15].

We next used conjFDR to identify specific genetic loci jointly associated with each mental disorder and risk phenotype. Through leveraging the cross-trait enrichment to boost statistical power, this enabled the identification of 98 novel risk loci in BIP and 38 in SCZ, Although these findings require further validation [36, 47]. A more complete understanding of the genomic architecture of SCZ and BIP is necessary to aid the translation of genetic research into clinical practice through more accurate polygenic risk scores and better defined neurobiological targets [80, 81]. We also identified 68 loci that were common to risk-taking and risky behaviours, thus increasing the validity of these findings. This approach was also utilised in the original risk-taking GWAS given the limitations of using single-item questionnaire measures [33].

We functionally annotated all jointly associated loci to explore putative biological mechanisms linking the polygenic overlap and phenotypic associations observed between risk-taking, BIP and SCZ. GABAergic pathways were implicated in both disorders via the several GABA-related gene-sets for BIP and risky behaviours, and *GABR(A)* gene linked to SCZ and both risk phenotypes, in line with findings in the original risk-taking GWAS [33]. It is also notable that *CACNA1C* was mapped to lead SNPs from all four analyses, while *CADM2* was mapped to lead SNPs from BIP and both risk-taking and risky behaviours. Interestingly, both genes were also implicated in a recent GWAS of impulsivity and drug experimentation [74]. Additionally, lead SNPs in the shared loci from all four analyses were significantly associated with altered gene expression in the caudate nucleus, nucleus accumbens, putamen, amygdala and hippocampus, anterior cingulate cortex and frontal cortex, among others. The finding of significant differential expression in the basal ganglia is particularly interesting given evidence of increased functional connectivity in the nucleus accumbens and increased striatal activity on task-based fMRI in risk-taking adolescents [82, 83]. More broadly, these regions mirror neuroimaging and electro-encephalogram studies which implicate the frontal cortex, the anterior cingulate cortex and the striatum in risk-taking in healthy volunteers [84, 85], SCZ [26, 30] and BIP [24]. Interestingly, with the addition of the amygdala and the hippocampus, these structures make up the frontal-striatal reward system circuitry [86]. Taken with experimental evidence linking dysfunctional reward system processing and risk-taking in BIP [87] and SCZ [88], this offers a plausible neurobiological mechanism underlying the SNP associations reported.

Our study had several limitations. Firstly, MiXeR analysis was not sufficiently powered to accurately quantify the shared and unique components beyond maximum possible overlap. Larger samples are required to provide more precise estimates, which would enable comparison of the size of overlap between phenotypes. Secondly, this analysis was using European samples only. It is essential that more diverse samples and improved methods for transancestral analysis are developed to widen the applicability of genetic studies. Thirdly, the single yes/no item used to measure risk-taking in the UKB cohort has limited reliability and construct validity and has rarely been used in the context of SCZ and BIP. In particular, it is possible that responses to this item were confounded by concurrent affective symptoms, concurrent substance use and history of mental health diagnosis. Nonetheless, the prevalence of BIP, SCZ and depressive symptoms were low in the risk-taking sample, minimising the effect of these potential confounders, this questionnaire measure correlates highly with a variety of distinct risk-related behaviours [50–54], and the simplicity of the item enabled the collection of a substantial sample size. We also focused our discussion on findings replicated across both risk phenotypes, which are likely to represent more valid findings. Fifthly, the risky behaviours phenotype was constructed using four distinct phenotypes, two of which were related to substance use. In addition to risk-taking, addictive behaviours also correlate with disinhibited personality types, conduct disorder and attention-deficit hyperactivity disorder, all three of which are in turn linked to the latent factor “externalising”. It is therefore important to note that the phenotypic and genetic correlation between risk-taking and risky behaviour may be interpreted as a correlation between two related constructs rather than capturing the same underlying risk-taking construct. Finally, functional annotation of highly polymorphic genetic loci may be unstable. We therefore conducted several functional analyses using independent datasets, including FUMA, ConsensusPathDB and BrainSpan to triangulate these findings.

In summary, our findings reveal extensive polygenic overlap between risk phenotypes and mental disorders with implications for how the polygenic architecture of complex disorders are conceptualised. We also identify specific loci underpinning this overlap, including 38 novel SCZ loci, 98 novel BIP loci and 68 loci common to both risk phenotypes. Functional annotation offered insights into neurobiological mechanisms underpinning the phenotypic overlap between BIP, SCZ and risk-taking, highlighting convergent roles for GABAergic systems, neuronal structure and function and structures implicated in the fronto-striatal reward system. Future work is required to better delineate the molecular genetic mechanisms underlying these statistical associations, and determine their interaction with other psychiatric disorders.

## Supplementary information

Supplementary Material

Supplementary Tables
