# Supplementary material for: Characterising the shared genetic determinants of bipolar disorder, schizophrenia and risk-taking
Source: Transl Psychiatry. 2021 Sep 8;11:466. doi: 10.1038/s41398-021-01576-4 (PMC8426401; doi:10.1038/s41398-021-01576-4)
Supplement: Supplementary file 1 — Supplementary Material [file 41398_2021_1576_MOESM1_ESM.doc]

# Supplementary Material: Characterising the shared genetic determinants of bipolar disorder, schizophrenia and risk-taking

**Supplementary figures 2-8**

**Supplementary methods 9-12**

**Supplementary results 13**

**References 14-15**

# Supplementary figures


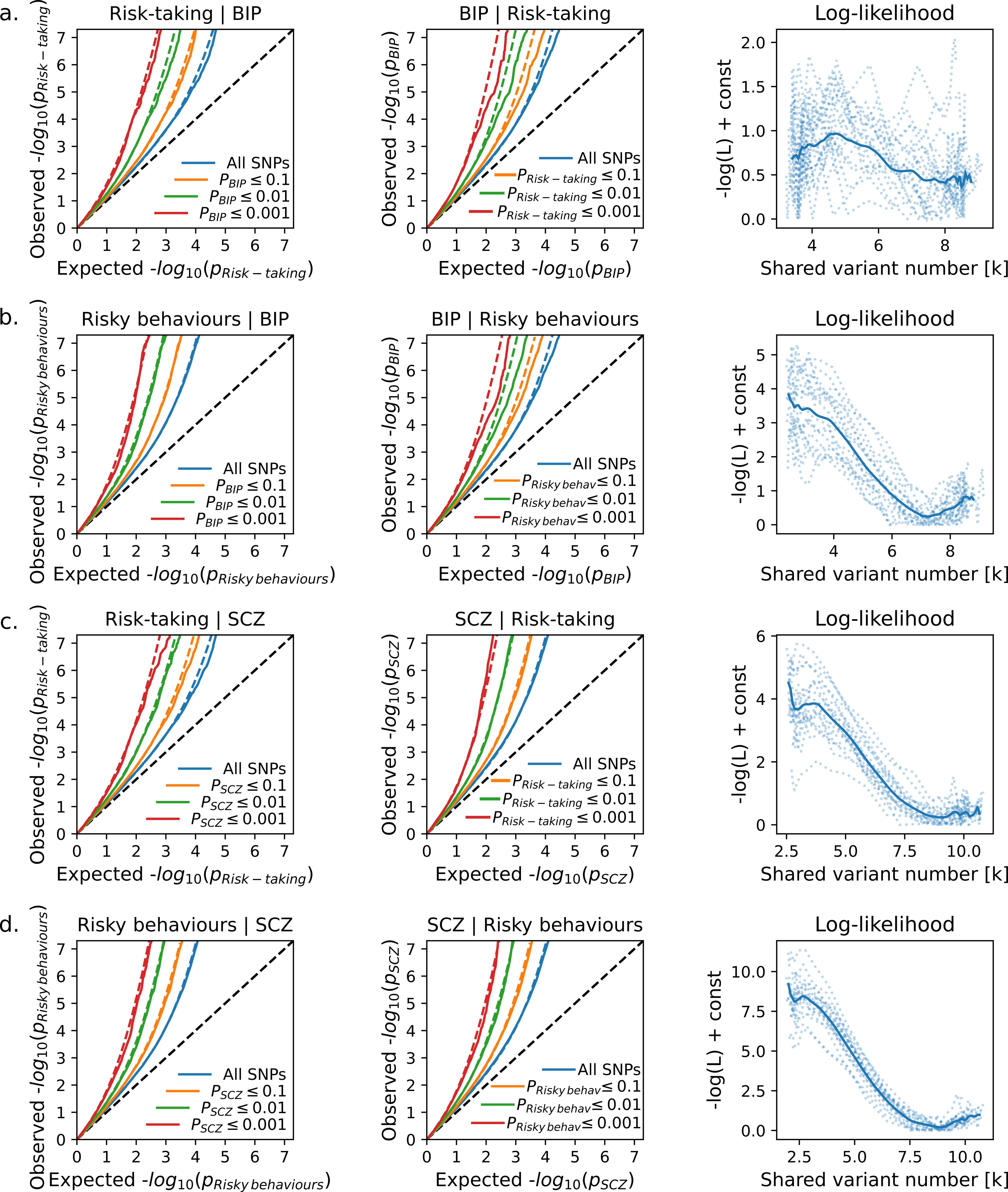


Supplementary figure 1: Supplemental MiXeR figures for a. BIP and risk-taking, b. BIP and risky behaviours c. SCZ and risk-taking and d. SCZ and risky behaviours. i. Conditional QQ plots of observed versus expected -log_10_ p-values in the primary trait as a function of the significance of the association with the secondary trait at the level of p ≤ 0.1 (orange lines), p ≤ 0.01 (green lines) and p ≤ 0.001 (red lines). Blue lines indicate all SNPs. Dotted lines indicate model predictions for each stratum. Black dotted line is the expected Q-Q plot under the null hypothesis (no SNPs associated with the phenotype). Points on the Q-Q plot are weighted according to LD structure, using n=64 iterations of random pruning at an LD threshold r^2^ = 0.1. ii. Likelihood-cost of the MiXeR Model. Log-likelihood of the bivariate fit as a function of 𝜋 parameter. The remaining parameters of the model were constrained to their fitted values.

A.
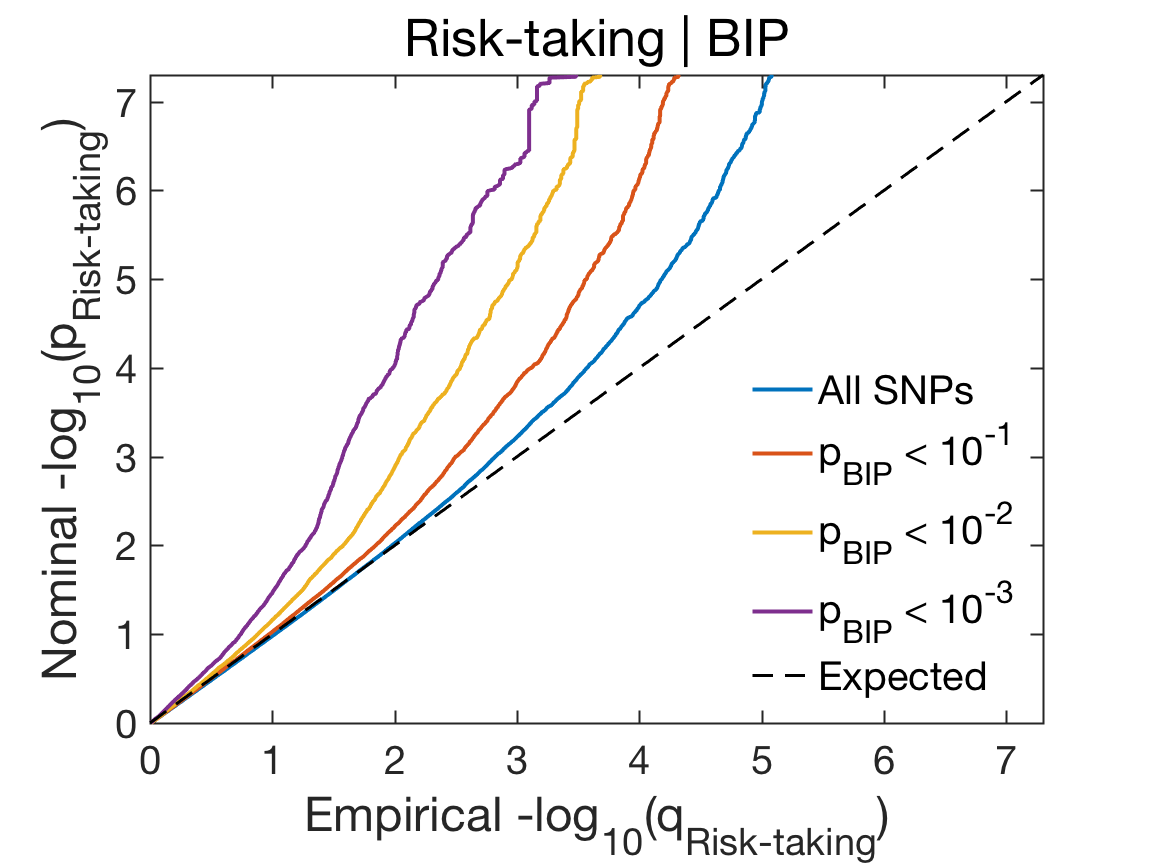
B.
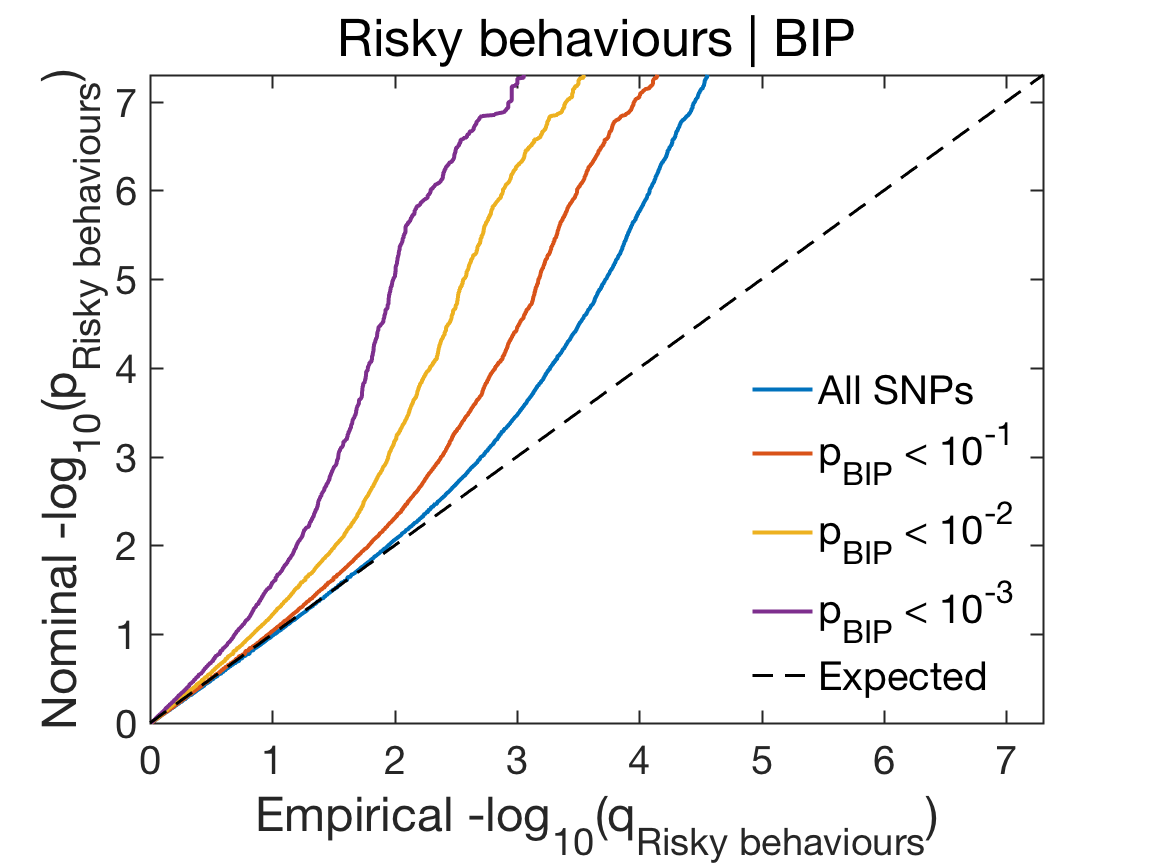


C.
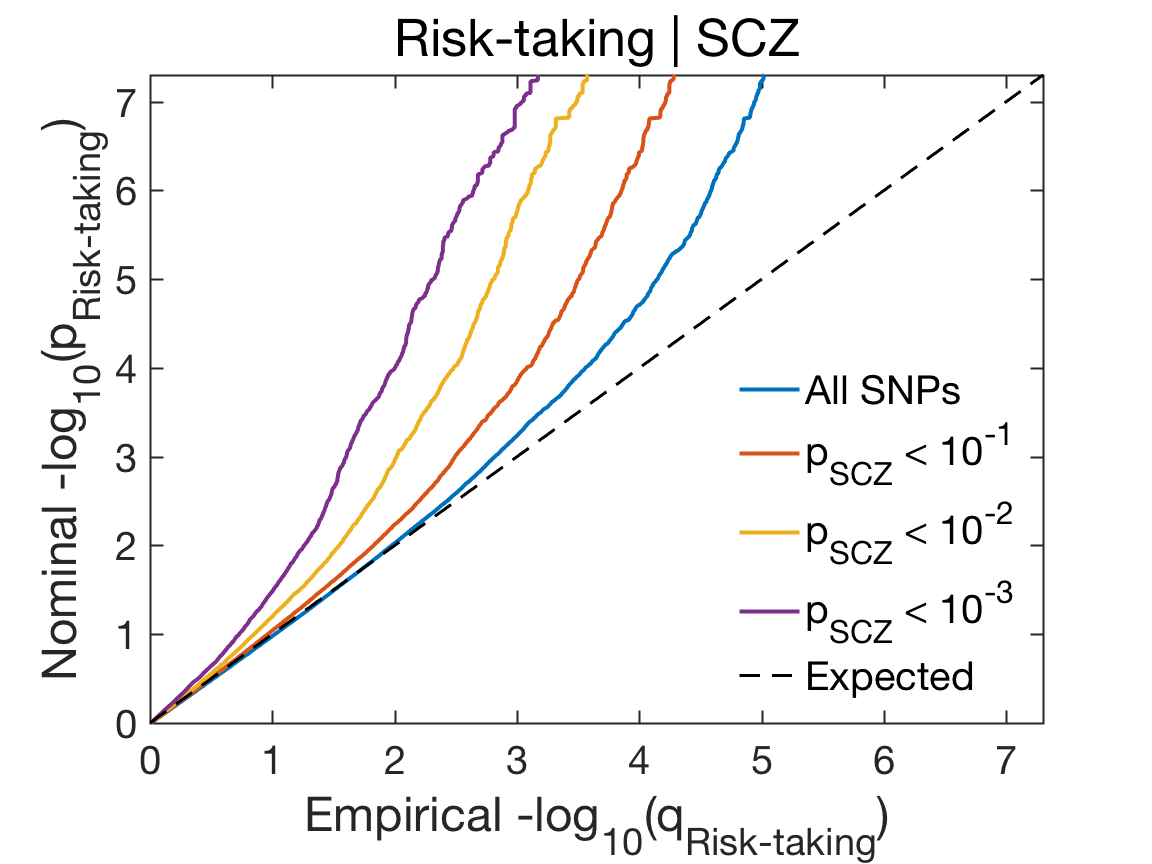
D.
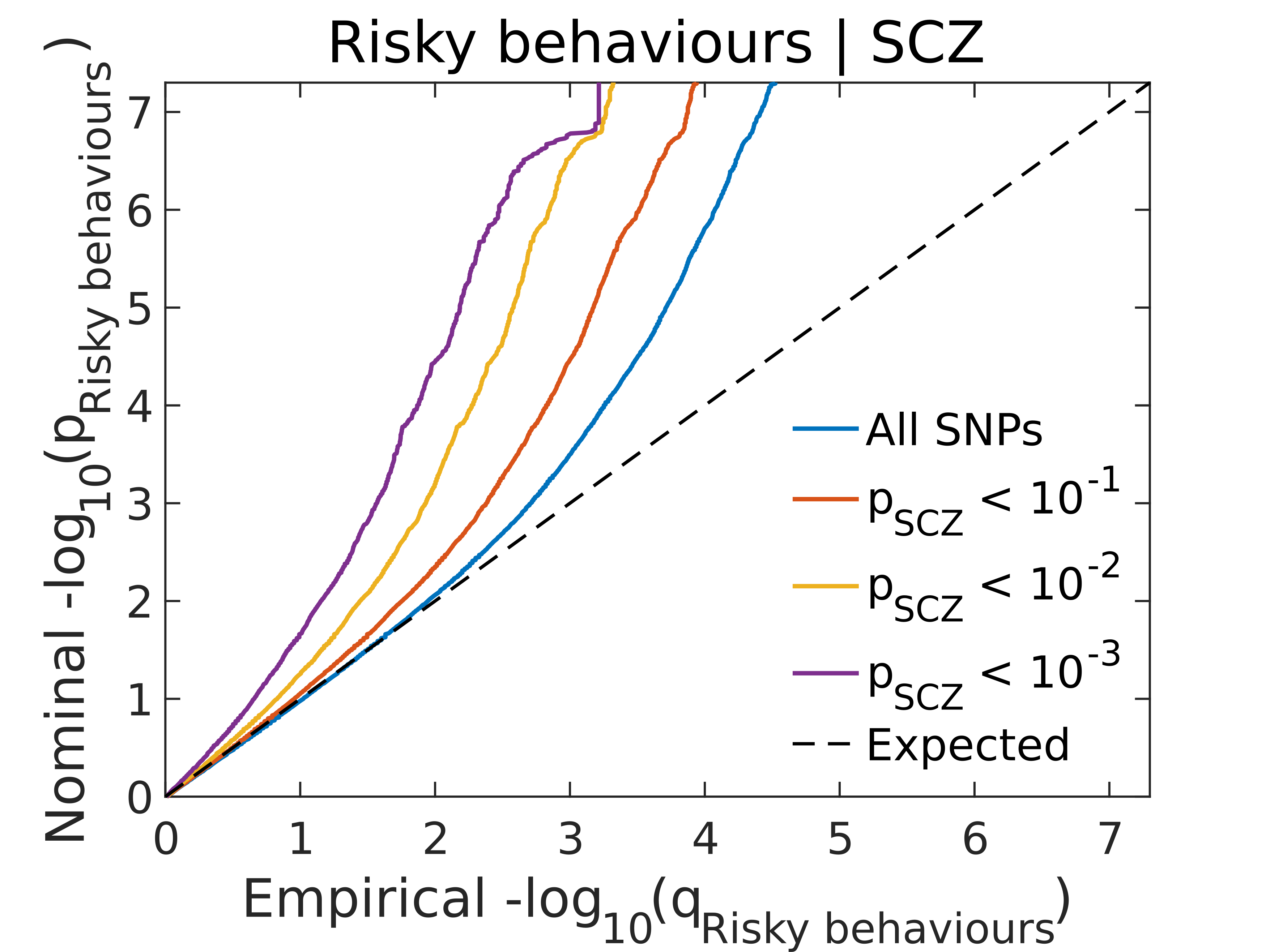


Supplementary figure 2: Reverse conditional Q-Q plots illustrating polygenic enrichment of SNPs associated with **a/c**. risk-taking and **b/d.** risky behaviours dependent on their association with **a-b.** BIP and **c-d.** SCZ. Conditional Q-Q plots of nominal versus empirical -log^10^p values in **a/c.** risk-taking or **b/d.** risky behaviours above the threshold of p<5x10^-8^ as a function of significance of association with **a-b.** BIP and **c-d.** SCZ at the level of p<0.1, p<0.01 and p<0.001 respectively.


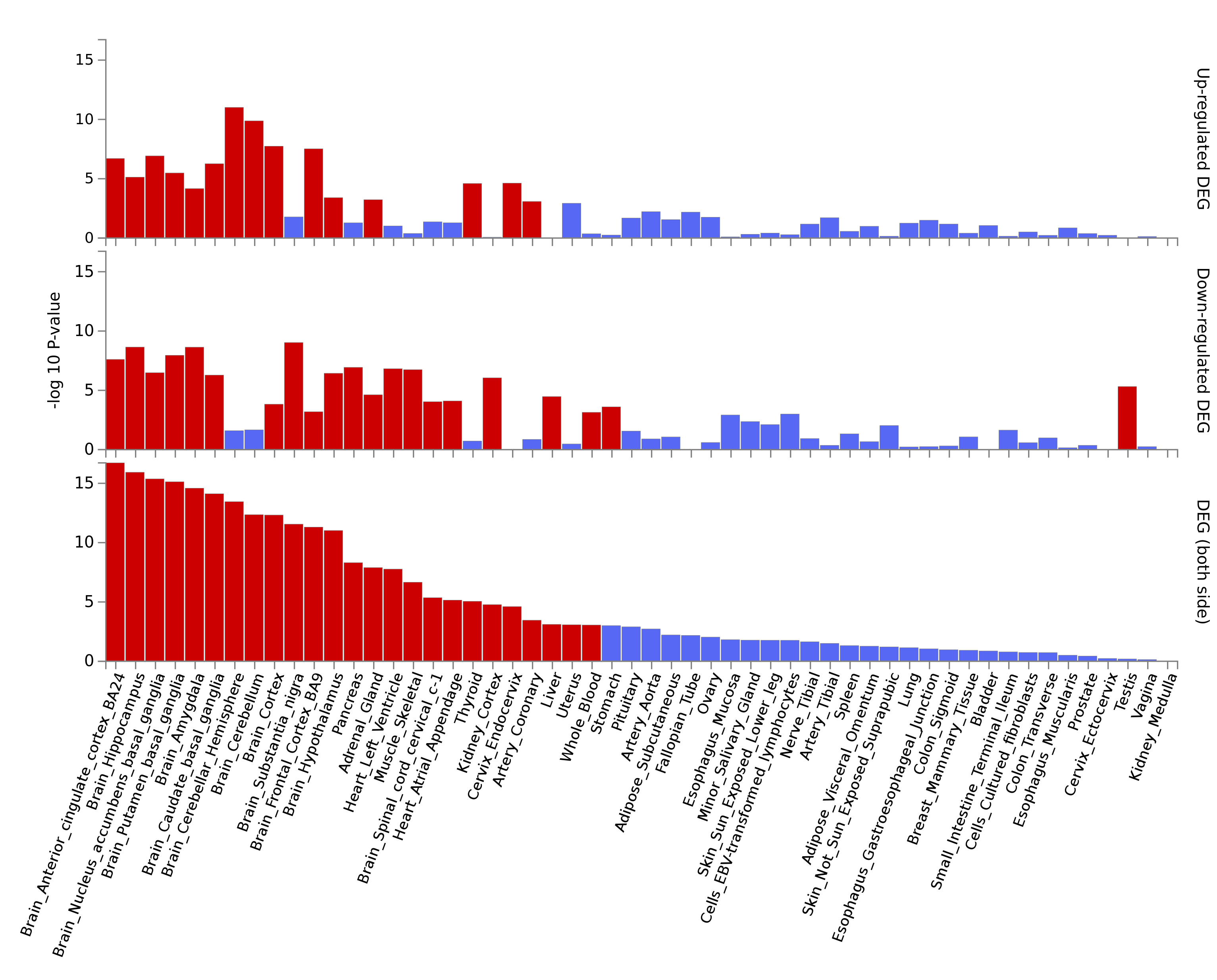


Supplementary figure 3: Differential expression of genes by tissue for the genes annotated to all lead SNPs conjunctionally associated with BIP and risk-taking at conjFDR<0.05. Red bars indicate statistical significance after correcting for multiple-testing.


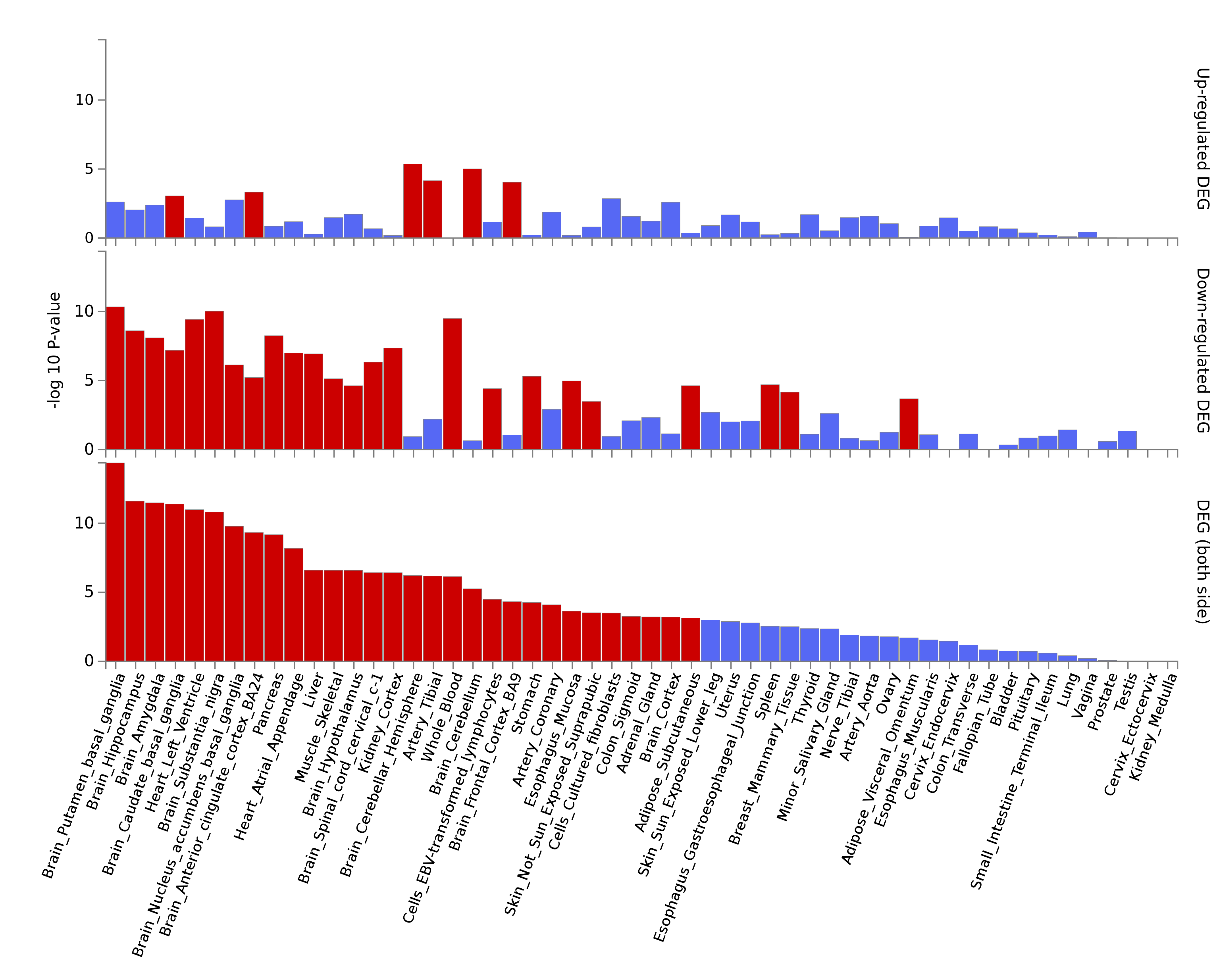


Supplementary figure 4: Differential expression of genes by tissue for the genes annotated to all lead SNPs conjunctionally associated with BIP and risky behaviours at conjFDR<0.05. Red bars indicate statistical significance after correcting for multiple testing.


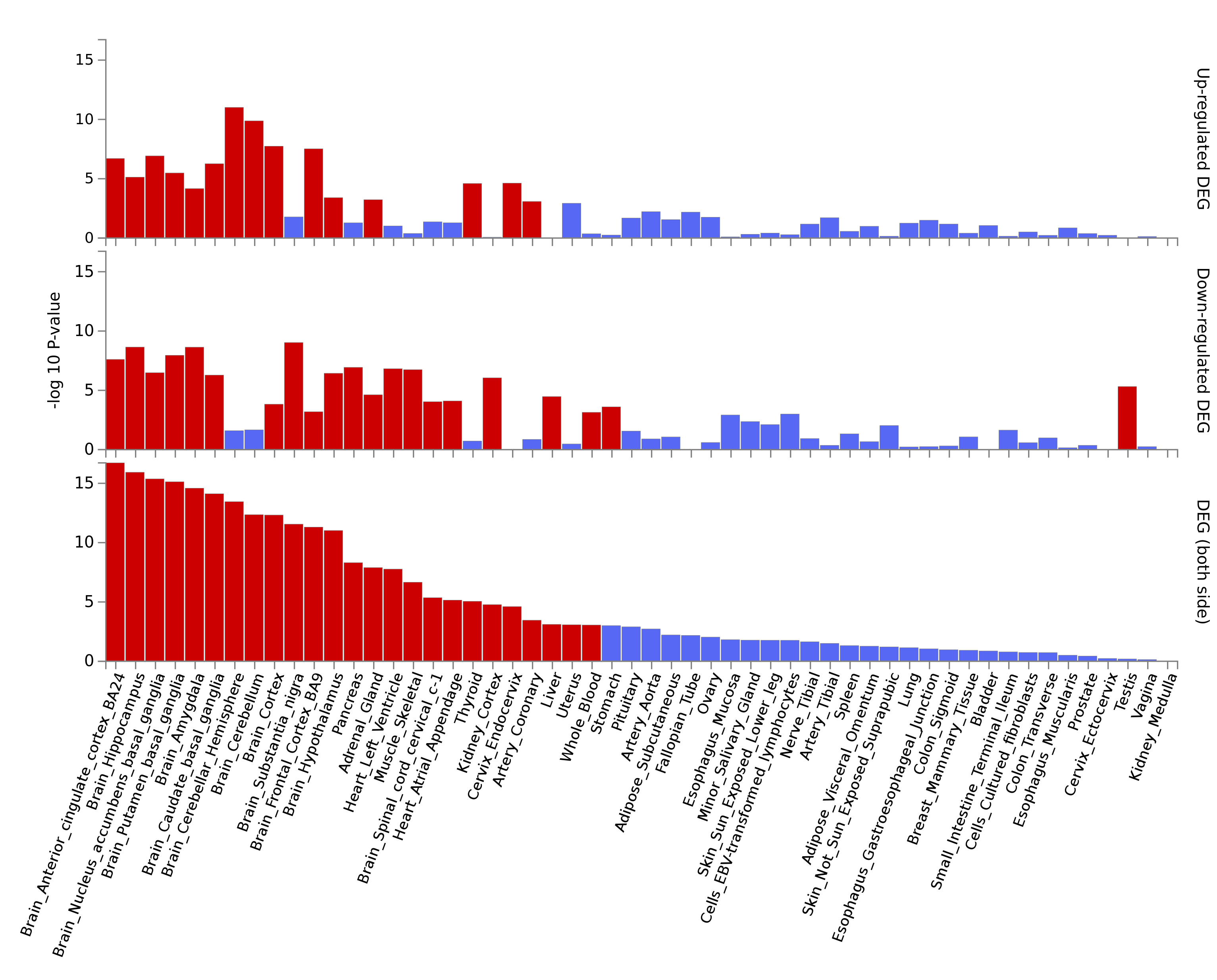
 Supplementary figure 5: Differential expression of genes by tissue for the genes mapped to all lead SNPs conjunctionally associated with SCZ and risk-taking at conjFDR<0.05. Red bars indicate statistical significance after correcting for multiple testing.

Supplementary figure 6: Differential expression of genes by tissue for the genes mapped to all lead SNPs conjunctionally associated with SCZ and risky behaviours at conjFDR<0.05. Red bars indicate statistical significance after correcting for multiple testing.

A.
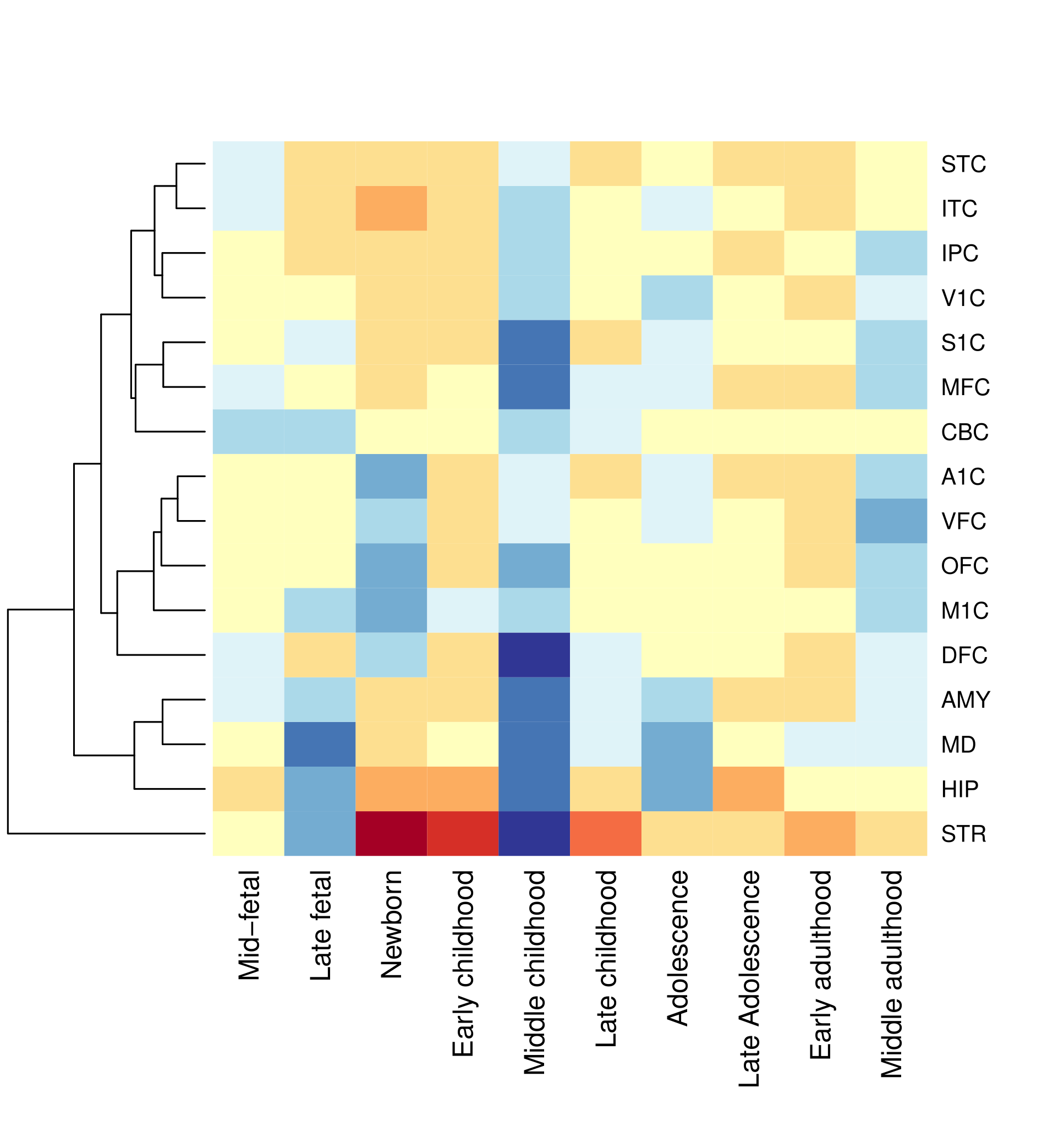
B.
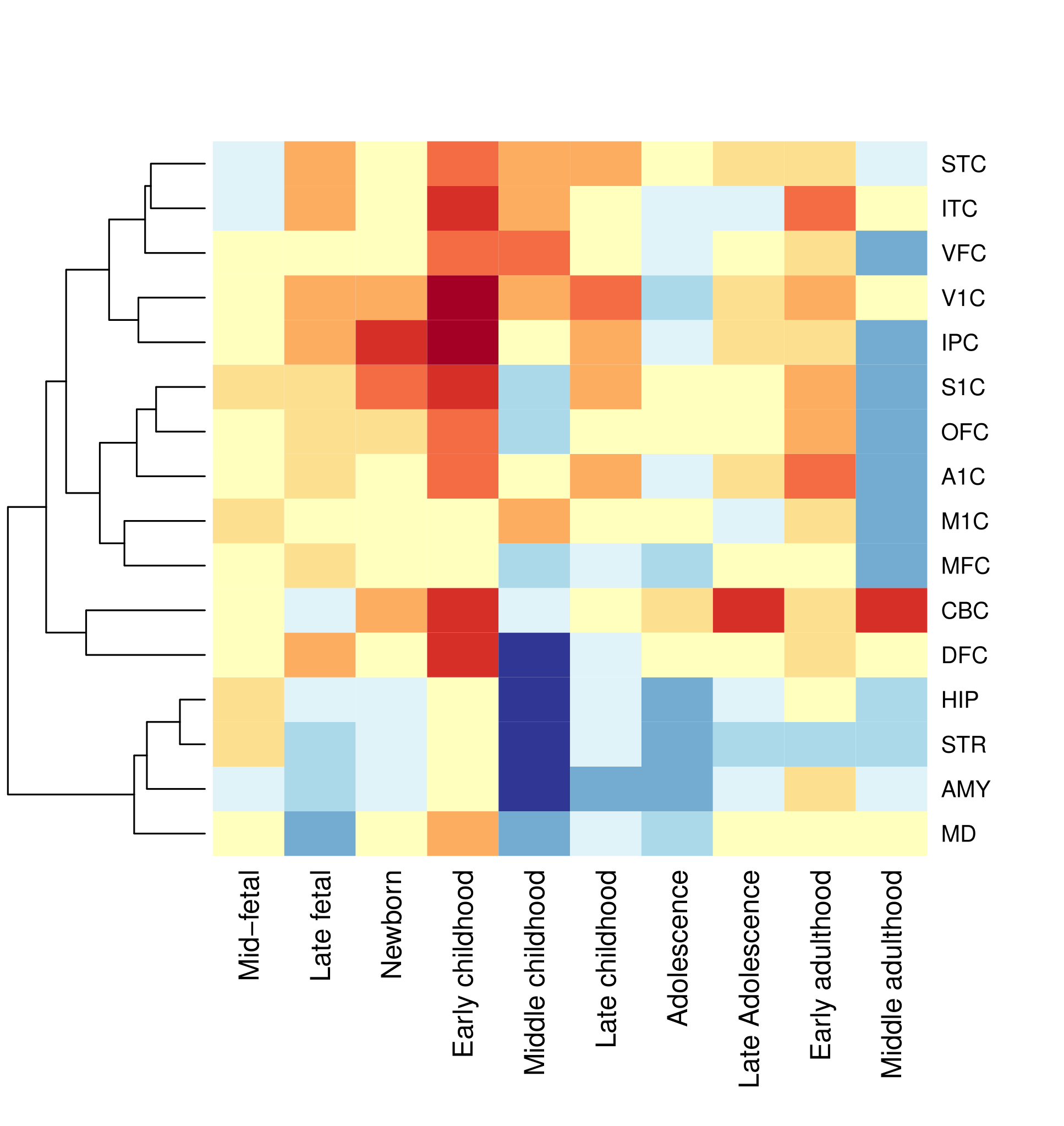


C.
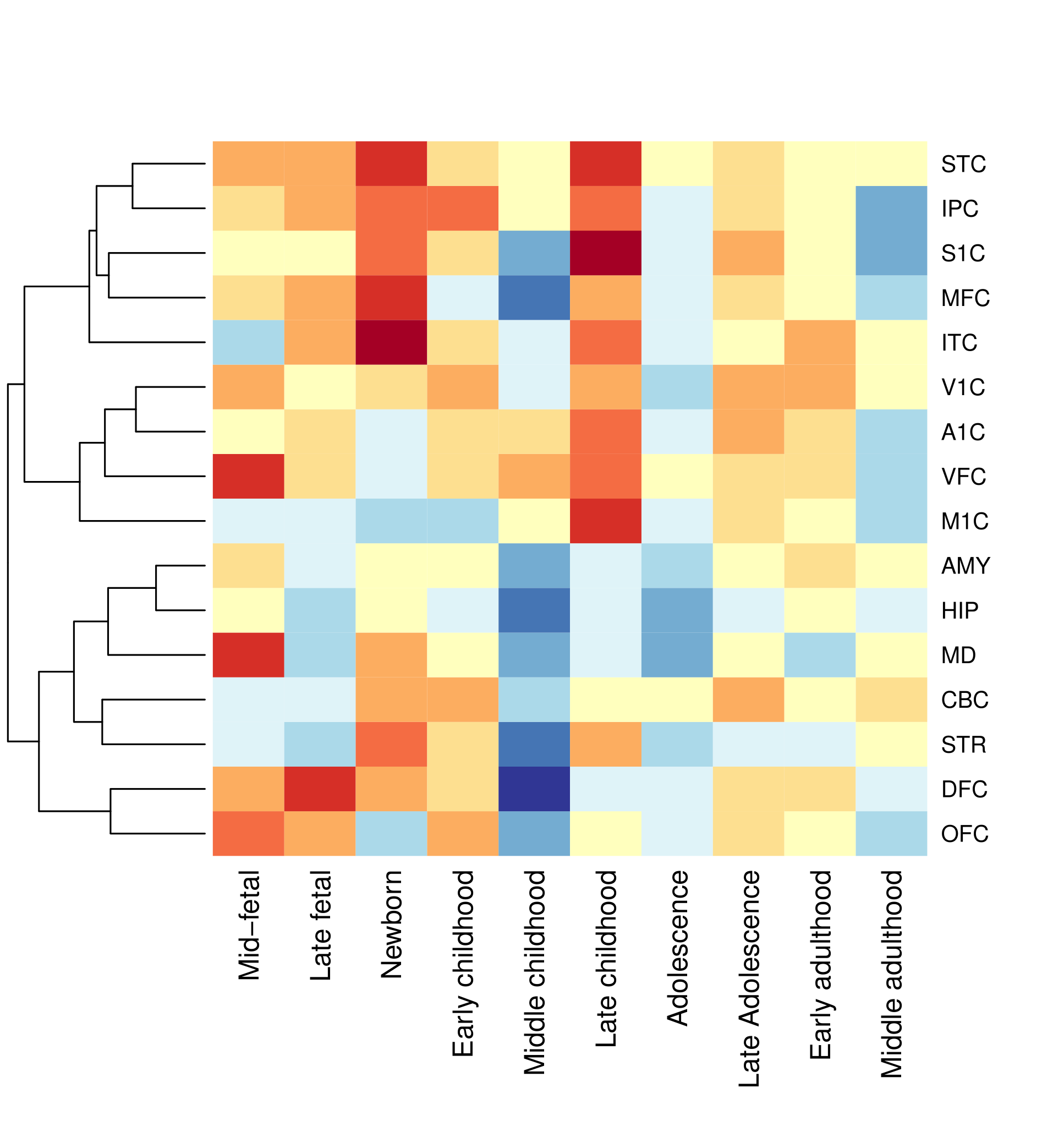
 D.
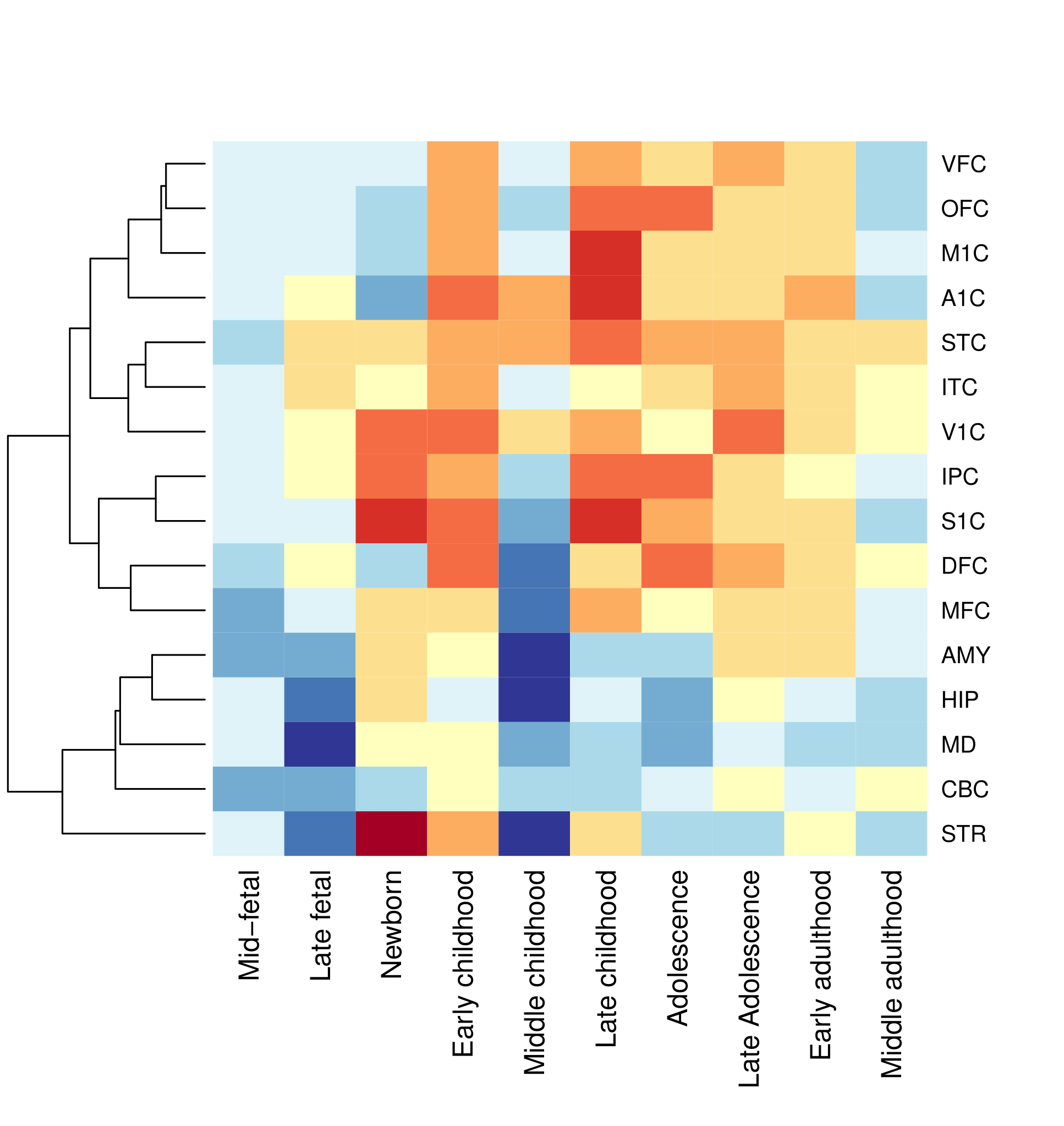


***Supplementary figure 7:*** ***Dendrogram and heat-map showing spatiotemporal gene expression of all mapped genes for a. risk-taking and bipolar disorder, b. risky behaviours and bipolar disorder, c. risk-taking and schizophrenia and d. risky behaviour and schizophrenia using RNA sequencing data from BrainSpan over 11 developmental periods (columns) and 16 brain regions (rows).*** *Gene expression is indicated from high (red) to low (blue). IPC = inferior parietal cortex, MFC = medial prefrontal cortex, STR = striatum, STC = superior temporal cortex, AMY = amygdala, CBC = cerebellum, HIP = hippocampus, DFC = dorsolateral prefrontal cortex, A1C = primary auditory cortex, S1C = primary sensory, M1C = primary motor cortex, V1C = primary visual cortex, ITC = inferior temporal cortex, VFC = ventrolateral prefrontal cortex, OFC = orbitofrontal cortices, THA = thalamus.*


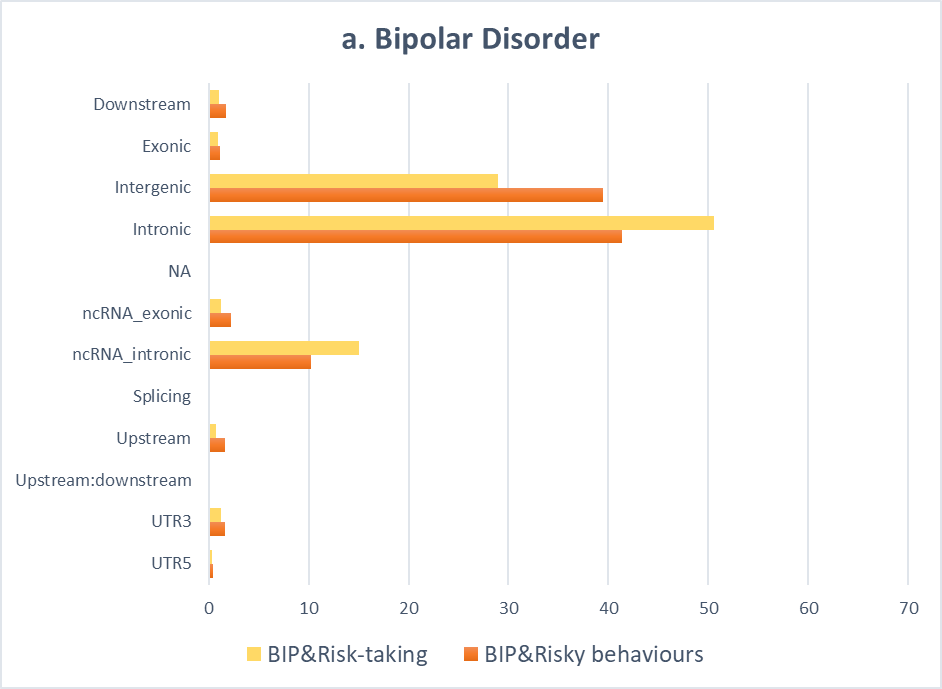

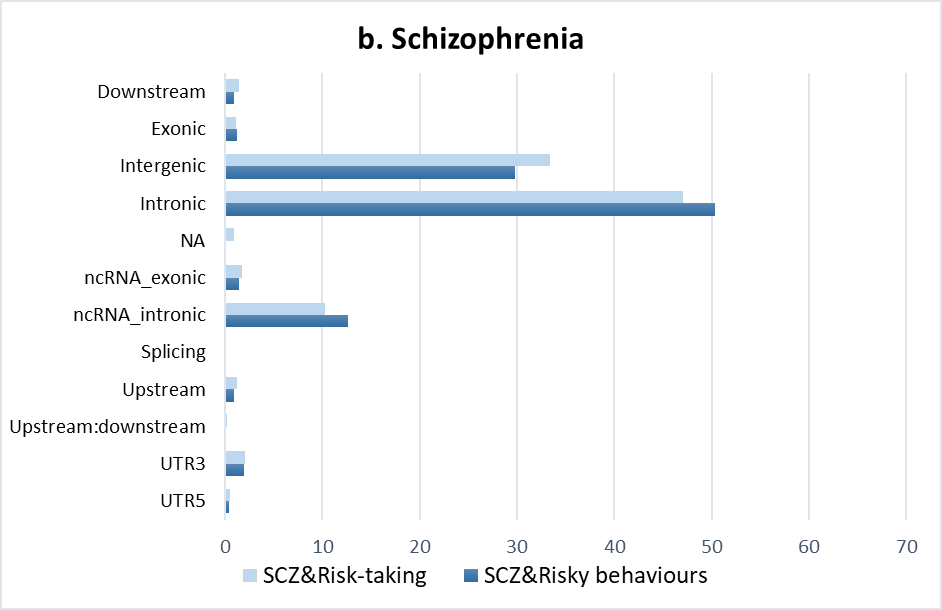


Supplementary figure 8: Bar chart illustrating the functional category of all candidate SNPs conjunctionally associated with a. bipolar disorder (BIP) and each of risk-taking and risky behaviours and b. schizophrenia (SCZ) and each of risk-taking and risky behaviours.

# Supplementary methods

## Bipolar sample

The majority of cases had BIP type I (n=25,060) while the remainder had either BIP type II (n=6,781), schizoaffective disorder bipolar type (n=977) or unspecified BIP (n=9099) [1].

## Risk-taking sample

Risk-taking was referred to as “risk tolerance” in the original GWAS. We refer to this as risk-taking to more accurately reflect the wording of the measure.

The 10 individual cohorts that made up the remaining 35,445 participants besides the UKB sample used different ordinal scales to measure risk-taking. These are described in detail in the original publication.[2]

The primary GWAS findings for this trait were well replicated in a second cohort using similar but distinct measures (genetic correlation = 0.83, s.e. = 0.13) and has significant genetic overlap with measures of specific risky behaviours such as substance misuse and propensity to drive over the speed limit [2].

## First principal component of four risky behaviours

The first principal component (PC) of four risky behaviours phenotype was acquired from the primary risk-taking GWAS publication which used UK Biobank data (*n* = 315,894). Automobile speeding propensity phenotype was derived from the item ‘How often do you drive faster than the speed limit on the motorway?’. Possible responses ranged from ‘(1) never/rarely’ to ‘(4) most of the time’. Respondents who answered ‘(5) do not drive on the motorway’ were excluded, after which the variable was normalised for male and females separately. The drinks per week phenotype was derived from responses to questions about drinking habits and was defined as the number of alcoholic drinks consumed per week. The ever-smoker phenotype in the UKB is a dummy variable that was coded one if a participant indicated that they were a current or previous smoker and zero if the participant reported never smoking or only smoking once or twice. The number of sexual partners phenotype was based on responses to the item ‘About how many sexual partners have you had in your lifetime?’; participants who indicated more than 99 lifetime sexual partners were required to confirm this response. Participants who reported that they had never had sex were coded 0, and the measure was also normalised separately for males and females. The first PC phenotype was then calculated as the first PC from a PC analysis of these four risky behaviours.

## MiXeR analysis

The MiXeR tool applies causal mixture models to GWAS summary statistics (<https://github.com/precimed/mixer>) [3,4]. For each SNP,
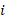
, univariate MiXeR models its additive genetic effect of allele substitution,
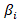
, as a point-normal mixture,
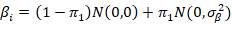
, where
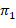
 represents ‘polygenicity’ (proportion of non-null SNPs) and
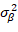
 represents ‘discoverability’ (variance of effect sizes of non-null SNPs). Then, for each SNP,
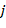
, MiXeR incorporates LD information and allele frequencies for M=9,997,231 SNPs extracted from 1000 Genomes Phase3 data by LD score regression software [5], and estimates the expected probability distribution of the signed test statistic,
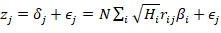
, where
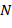
 is sample size,
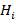
 is heterozygosity of i-th SNP,
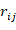
 is allelic correlation between i-th and j-th SNPs, and
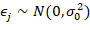
 is the residual variance. Further, the three parameters,
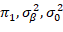
, are fitted by direct maximization of the likelihood function. The number of causal variants is estimated as
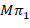
, where M = the number of SNPs in the reference panel (9,997,231 for the present analysis).

In the bivariate analysis, MiXeR models additive genetic effects as a mixture of four components: 1) null SNPs in both traits (
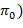
; SNPs affecting either 2) the first or 3) the second trait (
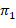
 and
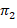
, respectively); and 4) SNPs with non-zero effect on both traits (
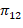
). In the shared component, MiXeR models the variance-covariance matrix as
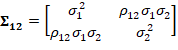
 where
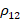
 indicates correlation of effect sizes within the shared component, and
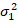
 and
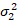
 correspond to the discoverability parameter estimated in the univariate analysis of the two traits. After fitting the parameters of the model, the genetic correlation is calculated as
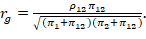
 Further information is available in [3].

A cut-off of 90% SNP heritability is chosen to avoid extrapolating the model parameters into SNP associations with infinitesimally small effect-sizes.

Univariate and bivariate estimates and standard errors were calculated by performing 20 iterations with 2 million randomly selected SNPs followed by random pruning at an LD threshold of r^2^=0.8, resulting in a sample of ~600K SNPs per iteration. The mean and standard error were then computed for each variable from the resulting sample of 20 iterations for each analysis.

To identify analyses using insufficiently powered GWAS summary statistics, we use the Akaike information criterion (
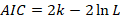
), where
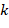
 is the number of free parameters in the model,
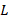
 is the value of the likelihood function, and
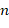
 is the effective number of SNPs used in the optimization procedure. We calculate the difference between
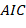
 between the full bivariate model,
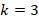
, and the reduced bivariate model,
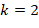
, due to
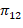
 being constrained to the smallest or largest possible value (
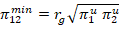
 and
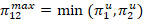
, respectively). A positive value of AIC indicates that the GWAS summary statistics have enough information to distinguish the custom polygenic overlap, as shown on the MiXeR Venn diagrams, versus the constrained models with minimal (
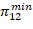
) and maximum (
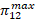
) polygenic overlap. This is visualised in the likelihood cost plots which plot negative log likelihood (y-axis) against the number of causal variants predicted (x-axis). The lowest point on the curve represents the “best” performing model, the furthest left point represents the minimum possible overlap and the furthest right represents the max possible overlap. Model-fit is also illustrated by predicted vs observed QQ plots. The closer the observed data follows the conditional-QQ plots, the better the model fits the data.#

## Conditional QQ plots

The data points on the QQ plot are weighted according to the LD structure around the corresponding SNP. We used n=200 iterations of random pruning with an LD threshold r2=0.1 to define LD-blocks throughout the genome. For each iteration, only one SNP from each block was selected to contribute to the p-value distribution statistics.

## Conjunctional FDR analysis

We re-ranked the test-statistics of our primary phenotypes (SCZ or BIP) according to the strength of the association with our secondary phenotypes (risk-taking and risky behaviours). This enables the estimation of the posterior probability that a given SNP is not associated with the primary trait given that its p-values are lower than the observed p-value for both the primary and secondary traits (conditional FDR value). The primary and secondary phenotypes were reversed, defined as the inverse conditional FDR value. The maximum of these two values represents a conservative estimate of the FDR for the association with both primary and secondary traits, defined as the conjFDR value, and can be interpreted as the strength of association between a given SNP and both traits [6]. We excluded SNPs in the major extended histocompatibility complex (MHC) and 8p23.1 regions from the FDR fitting procedures because their complex LD is liable to bias FDR estimation [36, 55].

## Genomic locus definition

Due to linkage disequilibrium, significantly associated SNPs were clumped into discrete “genomic loci” according to their cond/conjFDR value, the LD structure of approximate SNPs (quantified by the r^2^ value) and physical proximity. A given locus includes: a **lead SNP** - the most significantly associated SNP within a locus; **independent significant SNPs** - significant SNPs (cond/conjFDR<0.05) which are not in LD with each other (r2<0.6) but physically approximate (<250kb apart); and **candidate SNPs** which are in LD with independent significant SNPs (r^2^≥0.6) and have evidence of association with the primary trait or traits (conjFDR<0.1). In line with previous literature, the candidate SNP conjFDR threshold is lowered to maximise the probability that putative “causal” SNPs are captured within each locus.[7]

LD information was calculated from the 1000 genomes project reference panel [8].

## Functional annotation

We functionally annotated all candidate SNPs using the following strategies: (a) the Combined Annotation Dependent Depletion scores (CADD) which predicts how deleterious the SNP effect is on protein structure/function [9] (b) RegulomeDB scores which predict the likelihood of the SNP possessing a regulatory function [10] (c) the minimum chromatin state across 127 tissues at a given SNP locus. Lower scores are predictive of transcription or regulatory effects. [11] [12] (d) the genotype tissue expression (GTEx) resource which provides the association between genetic variants and gene expression levels across 44 tissue types, presented as the expression quantitative trait locus (eQTL) [13] (e) the UK Brain Expression Consortium (Braineac) which provides eQTL data from up to twelve brain regions from 134 brains free of neurodegenerative disorders [14] and (f) the Common Mind Consortium whose database contains eQTL data from approximately 1000 post-mortem brain samples from donors with SCZ, BIP and individuals without neuropsychiatric disorders across multiple regions [15].

We also constructed spatiotemporal heatmaps of gene expression levels across 11 brain tissues at 11 developmental timepoints in the R package “cerebroViz” using BrainSpan RNA sequencing data [16–18]. Expression across brain tissues was clustered using unsupervised hierarchical cluster analysis. The specific time points used were 16 weeks in utero (mid-fetal), 37 weeks in utero (late fetal), 4 months (Newborn), 1 year (Infancy), 3 years (Early childhood), 8 years (Middle childhood), 13 years (Late childhood), 19 years (Adolescence), 21 years (Late adolescence), 30 years (Early adulthood), and 36 years (Middle adulthood) [16–18].

# Supplementary results

**Sample demographics and phenotypic analyses**

Demographic and relevant clinical data for the primary risk-taking measure is provided in supplementary table 16 (below). Risk-taking was associated with decreased age, male sex, bipolar diagnosis, depression diagnosis, low mood, anhedonia, current daily smoking, past daily smoking, current daily alcohol and past daily alcohol but not schizophrenia. Despite this, the proportion of the total sample with history of mental disorders and current affective symptoms was low, and the differences between risk-taking groups were minimal. There were larger proportions of individuals with a current and past history of susbstance use was higher, and there were more substantial differences between risk-taking groups. However, this is not unexpected given the correlation between propensity to take risks and substance use. The implications of these findings are discussed in the limitations section of the discussion.

*Supplementary table 16: Demographic measures, history of mental disorder, concurrent symptoms of depression and substance use in the complete UKB sample by response to the primary risk-taking item. Complete-case analysis conducted since missing values <5% of total sample.*

|  | **Risk-taking** | | **Test statistic, p-value** |
| --- | --- | --- | --- |
|  | **Yes – n (% yes)** | **No – n (% no)** |
| **Total** | 129,839 (100) | 352,210 (100) | N/a |
| **Age** – mean (SD) | 55.52 (8.24) | 56.94 (7.99) | t=-54.22, p<2.23e-308 |
| **Sex** – female | 52,715 (40.60) | 208,929 (59.32) | χ^2^ = 13,394, p<2.23e-308 |
| **Diagnosis**  Bipolar  Schizophrenia  Depression | 515 (0.40)  161 (0.12)  8,185 (6.30) | 881 (0.25)  429 (0.12)  21,535 (6.11) | χ^2^ = 71.3, p=3.26e-16  χ^2^ = 1.08, p=0.58  χ^2^ = 56.1, p=6.42e-13 |
| **Current affective symptoms**  Low mood nearly every day  Anhedonia nearly every day | 2,916 (2.25)  3,517 (2.71) | 6,023 (1.71)  6,409 (1.82) | χ^2^ = 149.35, p=2.41e-34  χ^2^ = 371.4, p=9.28e-83 |
| **Substance use**  Current daily smoking  Past daily smoking  Current daily alcohol  Former daily alcohol | 13,693 (10.55)  34,611 (26.66)  31,692 (24.41)  5,030 (3.87) | 23,632 (6.71)  81,885 (23.25)  66,727 (18.95)  12,348 (3.51) | χ^2^ = 1954.2 p<2.23e-308  χ^2^ = 601.0, p=1.01e-32  χ^2^ = 1742, p<2.23e-308  χ^2^ = 371.4, p=9.28e-83 |

**Mixer model fit**

Observed versus expected conditional-QQ plots demonstrated that there was adequate model-fit (*Supplementary figure 1)*. AIC analyses showed that MiXeR was sufficiently powered to differentiate estimated polygenic overlap from minimum possible overlap but insufficiently powered to differentiate it from maximum overlap. This implies that MiXeR was adequately powered to demonstrate extensive overlap between BIP, SCZ and risk phenotypes, but not sufficiently powered to provide precise estimates for the unique components. Larger GWAS samples are required to improve model precision.

**Further functional annotation**

Functional annotation of loci jointly associated with BIP and risk-phenotypes revealed 41.3% and 39.4% of all candidate SNPs for risk-taking and BIP (n=6877) were intronic and intergenic, respectively, while 1.1% where exonic. In contrast, 50.5% of candidate SNPs for risky behaviours and BIP (n=8405) were intronic, 28.9% were intergenic and 0.9% were exonic (*Supplementary figure 3a, Supplementary tables 4-5*).

Of the 7544 candidate SNPs associated with both SCZ and risk-taking, the majority were either intronic (47.1%) or intergenic (33.4%), while 1.2% were exonic (*Supplementary table 10, Supplementary figure 3b*). Similarly, 50.4% of the candidate SNPs for risky behaviours and SCZ were intronic, 29.8% were intergenic and 1.3% were exonic (n=7752, *Supplementary table 11*).

Spatiotemporal gene expression analysis showed highest gene expression of mapped genes for risk-taking and bipolar and risky behaviours and schizophrenia in the striatum during the newborn and early childhood periods, supporting the differential expression findings using independent gene expression data. All four analyses also demonstrated a distinctive reduction of gene expression of mapped genes during middle childhood. Spatiotemporal expression of mapped genes for bipolar and risky behaviours and risk-taking and schizophrenia was more heterogenous, with high expression across multiple brain regions during early childhood for bipolar and risky behaviours and during the newborn period and late childhood for schizophrenia and risk-taking.

**References**

1. Mullins N, Forstner AJ, O’Connell KS, Coombes B, Coleman JRI, Qiao Z, et al. Genome-wide association study of more than 40,000 bipolar disorder cases provides new insights into the underlying biology. Nat Genet. 2021. 17 May 2021. https://doi.org/10.1038/s41588-021-00857-4.

2. Karlsson Linnér R, Biroli P, Kong E, Meddens SFW, Wedow R, Fontana MA, et al. Genome-wide association analyses of risk tolerance and risky behaviors in over 1 million individuals identify hundreds of loci and shared genetic influences. Nat Genet. 2019;51:245–257.

3. Frei O, Holland D, Smeland OB, Shadrin AA, Fan CC, Maeland S, et al. Bivariate causal mixture model quantifies polygenic overlap between complex traits beyond genetic correlation. Nat Commun. 2019;10:1–11.

4. Holland D, Frei O, Desikan R, Fan C-C, Shadrin AA, Smeland OB, et al. Beyond snp heritability: Polygenicity and discoverability of phenotypes estimated with a univariate gaussian mixture model. BioRxiv. 2019;16:133132.

5. Bulik-Sullivan BK, Loh P-R, Finucane HK, Ripke S, Yang J, Patterson N, et al. LD Score regression distinguishes confounding from polygenicity in genome-wide association studies. Nat Genet. 2015;47:291.

6. Andreassen OA, Djurovic S, Thompson WK, Schork AJ, Kendler KS, O’Donovan MC, et al. Improved detection of common variants associated with schizophrenia by leveraging pleiotropy with cardiovascular-disease risk factors. Am J Hum Genet. 2013;92:197–209.

7. Smeland OB, Shadrin A, Bahrami S, Broce I, Tesli M, Frei O, et al. Genome-wide association analysis of Parkinson’s disease and schizophrenia reveals shared genetic architecture and identifies novel risk loci. Biol Psychiatry. 2020. 2020.

8. Auton A, Abecasis GR, Altshuler DM, Durbin RM, Bentley DR, Chakravarti A, et al. A global reference for human genetic variation. Nature. 2015;526:68–74.

9. Rentzsch P, Witten D, Cooper GM, Shendure J, Kircher M. CADD: predicting the deleteriousness of variants throughout the human genome. Nucleic Acids Res. 2018;47:D886–D894.

10. Boyle AP, Hong EL, Hariharan M, Cheng Y, Schaub MA, Kasowski M, et al. Annotation of functional variation in personal genomes using RegulomeDB. Genome Res. 2012;22:1790–1797.

11. Kundaje A, Meuleman W, Ernst J, Bilenky M, Yen A, Heravi-Moussavi A, et al. Integrative analysis of 111 reference human epigenomes. Nature. 2015;518:317.

12. Ashburner M, Ball CA, Blake JA, Botstein D, Butler H, Cherry JM, et al. Gene Ontology: tool for the unification of biology. Nat Genet. 2000;25:25–29.

13. GTEx Consortium, Aguet F, Brown AA, Castel SE, Davis JR, He Y, et al. Genetic effects on gene expression across human tissues. Nature. 2017;550:204.

14. Ramasamy A, Trabzuni D, Guelfi S, Varghese V, Smith C, Walker R, et al. Genetic variability in the regulation of gene expression in ten regions of the human brain. Nat Neurosci. 2014;17:1418.

15. Fromer M, Roussos P, Sieberts SK, Johnson JS, Kavanagh DH, Perumal TM, et al. Gene expression elucidates functional impact of polygenic risk for schizophrenia. Nat Neurosci. 2016;19:1442–1453.

16. Miller JA, Ding S-L, Sunkin SM, Smith KA, Ng L, Szafer A, et al. Transcriptional landscape of the prenatal human brain. Nature. 2014;508:199–206.

17. BrainSpan. BrainSpan Atlas of the Developing Human Brain. 2010. http://www.brainspan.org/. Accessed 16 October 2020.

18. Bahl E, Koomar T, Michaelson JJ. cerebroViz: an R package for anatomical visualization of spatiotemporal brain data. Bioinformatics. 2017;33:762–763.
